# Hippocampal representations of foraging trajectories depend upon spatial context

**DOI:** 10.1038/s41593-022-01201-7

**Published:** 2022-11-29

**Authors:** Wan-Chen Jiang, Shengjin Xu, Joshua T. Dudman

**Affiliations:** 1 Janelia Research Campus, Howard Hughes Medical Institute, Ashburn, VA USA; 2 Present Address: Institute of Neuroscience, State Key Laboratory of Neuroscience, Center for Excellence in Brain Science and Intelligence Technology, Chinese Academy of Sciences, Shanghai, China

**Keywords:** Hippocampus, Learning algorithms, Neural circuits

## Abstract

Animals learn trajectories to rewards in both spatial, navigational contexts and relational, non-navigational contexts. Synchronous reactivation of hippocampal activity is thought to be critical for recall and evaluation of trajectories for learning. Do hippocampal representations differentially contribute to experience-dependent learning of trajectories across spatial and relational contexts? In this study, we trained mice to navigate to a hidden target in a physical arena or manipulate a joystick to a virtual target to collect delayed rewards. In a navigational context, calcium imaging in freely moving mice revealed that synchronous CA1 reactivation was retrospective and important for evaluation of prior navigational trajectories. In a non-navigational context, reactivation was prospective and important for initiation of joystick trajectories, even in the same animals trained in both contexts. Adaptation of trajectories to a new target was well-explained by a common learning algorithm in which hippocampal activity makes dissociable contributions to reinforcement learning computations depending upon spatial context.

## Abstract (web summary)

Model-based analysis of learning and neural activity in mice trained to find reward in both a spatial, navigational context and a relational, non-navigational context revealed dissociable contributions of hippocampus depending upon spatial context.

## Main

A critical problem for an animal, or, in general, an agent, is to learn how to forage for desired outcomes such as water or food (more generally, a ‘reward’). Learning how to refine behavior so as to reliably obtain reward through experience is the core question addressed by reinforcement learning (RL) theory^1^. The problem is particularly acute when rewards are sparse and/or distal—for example, problems requiring navigation to intercept a distant spatial reward location or when a specific, non-navigational action or sequence of actions is required for reward.

Neural activity in the dorsal hippocampus is thought to provide rich and flexible representations of recent experience critical for learned spatiotemporal associations among locations, stimuli and outcomes^2^. Neural activity in dorsal CA1 (dCA1), a key node in the hippocampal–entorhinal circuit, is organized at multiple spatiotemporal scales. Individual principal cells in dCA1 tend to be active in circumscribed regions of space (‘place cells’ with ‘place fields’)^3^ with a broadly distributed propensity for activation^4^ that allows for efficient representation of spaces differing by orders of magnitude^5^. On a time scale of seconds, ensembles of place cells are organized into brief sequential bouts of activation as an animal actively navigates through an environment^6^. Similar temporal patterns of activity are associated with non-spatial dimensions in tasks requiring an animal to associate distinct stimuli^7^, track changing sensory input^8,9^ or measure elapsed time^10,11^. In both spatial/navigational and non-spatial contexts, these representations on the order of seconds are proposed to reflect a cognitive map—a representation of the relations between states of the environment^12,13^.

When animals are not actively moving^14,15^ or are asleep^16^, large ensembles of dCA1 neurons burst in brief synchronous population events (SPEs) that occur for durations on the order of 150 ms and tend to be associated with sharp-wave ripples (SWRs) in the local field potential^17^. SPEs have been shown to reactivate populations of dCA1 neurons that were recruited during active navigation^14,15,18^ or states of active behavior while stimuli were encoded in non-spatial tasks^19^. In diverse memory tasks and species, perturbation experiments have implicated each of these components of the dCA1 spatiotemporal representation in aspects of learned behavior^20–23^. SWRs have been associated with functions ranging from memory recall^24^ to consolidation^22^ to decision-making^25^ and are thought to act as a compressed readout of an underlying cognitive map representation^19,26^.

Several studies have observed changes in the hippocampal–entorhinal representation of space in the vicinity of rewarded spatial targets^27–29^. This has led to influential proposals that the hippocampus represents a map of locations that may be used to store location of rewards^30^, and this map can be read out via SWR reactivation for planning of future reward-seeking actions or consolidation of recent experience^17,25^. Such models provide an elegant solution for the construction of cognitive maps representing reward either as a location in a spatial environment or a specific state in a non-spatial environment, suggesting the possibility of a universal computational role of hippocampal cognitive maps across contexts^13,31^. However, the spatial context of an environment can also specify unique behavioral requirements. Executing a navigational foraging trajectory and a non-navigational action sequence are distinct behaviors thought to depend upon largely separable brain circuits for their control. Thus, although it is clear that hippocampal representations in navigational and non-navigational contexts are similar^9,32^, to date it is much less clear how these representations can differ and whether they make distinct contributions to learning dependent upon behavioral demands.

These questions have been difficult to address because hippocampal activity, behavior and targeted manipulations of hippocampal function have not been directly compared in animals trained in distinct contexts (for example, navigational and non-navigational) but with shared relational structures (for example, execute a foraging trajectory, wait for reward feedback and update parameters of future trajectories). To overcome this barrier, we studied two tasks in which mice had to execute foraging trajectories to intercept a hidden reward target in distinct navigational and non-navigational contexts. Imaging of population activity in behaving mice revealed partially dissociable neuronal representations of foraging trajectories across spatiotemporal scales in dCA1. Despite the highly similar structure, the timing of task-related SPEs remapped from retrospective timing in a navigational context to prospective timing in a non-navigational context, even in the same animal trained in both contexts. We extend previous work to describe an RL algorithm that learns to adapt foraging trajectories in both contexts highly similar to those observed in animals. Using model-based analysis, we provide multiple lines of evidence that dCA1 SPEs are a key but context-dependent component of reinforced adaptations in foraging trajectories.

## Results

### Paired navigational and non-navigational foraging tasks

**Fig. 1:**
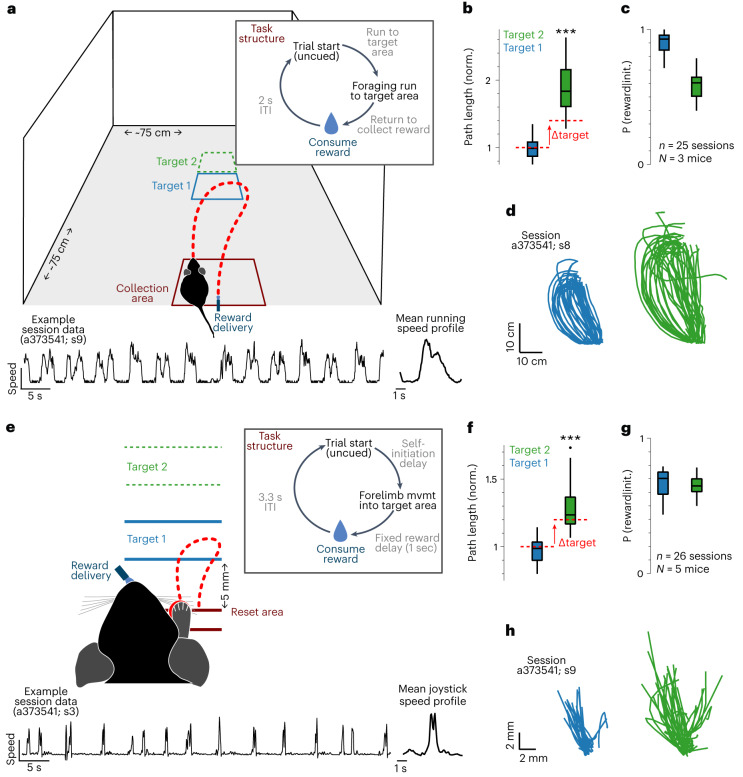
Navigational (STF) and non-navigational (NTF) foraging tasks. **a**, Schematic representation of the freely moving STF task. Upper right inset shows the trial structure. Lower traces show continuous running speed traces and average speed trace of multiple trials from an example session. Halfway through a session, the original target (1, blue) was moved to a more distal location (2, green) without any cue. **b**, Change in path length of foraging runs as a function of target distance in STF task (1: close (blue); 2: far (green)) for all mice and sessions. **c**, Performance was estimated as the probability of correctly intercepting the target given that a foraging attempt was initiated (Methods). Average performance for the two target locations across all animals and sessions (1: close (blue); 2: far (green)). *n* = 25 sessions and *N* = 3 mice in **b** and **c**. **d**, All trajectories that intercepted the target (1: close (blue); 2: far (green)) are shown for a representative STF session. **e**, Schematic of the head-fixed NTF task in which mice moved a joystick to a hidden target location. Brief occupancy of the target area (~100 ms) yields an unsignaled, delayed (1-second) water reward through a spout. Upper right inset shows the trial structure of the NTF task. Lower traces show continuous joystick speed traces and average speed trace of multiple trials from an example session. **f**, Path length of joystick trajectories in the NTF task (1: close (blue); 2: far (green)) for two blocks that switched from a near to a far target location. **g**, Performance was estimated as the probability of correctly intercepting the target given that a foraging attempt was initiated (Methods). Average performance for the two target locations across all animals and sessions (1: close (blue); 2: far (green)). *n* = 26 sessions and *N* = 5 mice in **f** and **g**. **h**, All trajectories that intercepted the target (1: close (blue); 2: far (green)) are shown for a representative NTF session from the same animal in **d**. Statistical testing: two-tailed rank-sum test, ***P < 0.001. Box plot properties: center: median; edges: 25th–75th percentiles; whiskers: extrema; outliers plotted individually.

First, we describe here a self-paced foraging task in which freely moving mice run to an unmarked target location tens of centimeters away from a reward collection area (spatial target foraging (STF) task; Fig. 1a–d and Supplementary Video 1). Second, we modified a head-fixed operant task^33^ in which a mouse must displace a spring-loaded joystick from a center position to a target distance on the order of 10 mm away from its central resting location (non-navigational^34^ target foraging (NTF) task; Fig. 1e–h). In both tasks, the delivery of reward was dissociated from movement into the target location that triggered reward. In the STF task, this was accomplished by delivering a reward via a water port at a specific ‘home’ location on a wall ~30 cm away from the target location. In contrast, in the NTF task, this was accomplished by delivering water reward with a 1-second delay after movement to the target area (forelimb movement duration ~0.5 seconds^35^). As the target location shifted, mice reliably scaled their movement trajectories to a similar relative extent in both tasks (Fig. 1b,f). Performance, assessed as the probability of collecting a reward given that a foraging attempt was initiated, was high in both tasks and exceeded 0.6 even for distant target locations (Fig. 1c,g). In both tasks, interception trajectories were variable in their angle and heading when the same animal was trained in both contexts (Fig. 1d,h).

### A common algorithm accounts for learning across task contexts

**Fig. 2:**
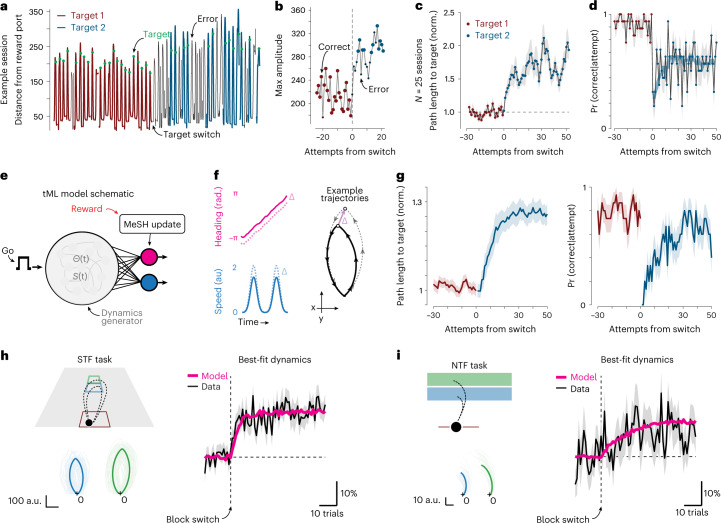
An RL account of trajectory changes after target switch in STF and NTF tasks. **a**, Example trajectories in STF are plotted as distance × time curves with intervals of immobility removed. The point of target interception is denoted by a green dot, and color intensity reflects whether a given trajectory attempt was correct (‘target’) or an error. **b**, Maximum amplitude of individual foraging trajectories aligned to the target switch in an example session. Large dots denote correct attempts, and small dots denote errors for a single session. **c**, Mean amplitude of foraging trajectory attempts aligned to target switch for all sessions (*n* = 25 sessions and *N* = 3 mice); red dot: target 1; blue dot: target 2. **d**, Average probability of a correct target interception per attempt aligned around the target switch. Smooth curve is a Savitzky–Golay filtered estimate. **e**, Schematic representation of the tML model (Methods). The foraging trajectory is governed by two dynamical outputs: heading (pink) and speed (cyan) simulated as functions {Θ(t) and S(t)} (Methods) as indicated and used for simulations herein. A learning rule is used to update the heading offset and speed scaling dependent upon performance feedback (‘reward’, red). **f**, Examples of the time-varying heading (pink) and speed signals (cyan) plus a learned shift (Δ). **g**, A simulation of the STF task was run exhibiting smooth adjustment of trajectory amplitude (left, compared to **c**) and probability of intercepting the target aligned to target switch (right, compared to **d**). **h**,**i**, The tML model was tested in a simulated version of the STF (**h**) and NTF (**i**) tasks. Left: top, a schematic of the task is shown with trajectories from an example simulation across the close (blue) and far (green) blocks; bottom, heavy line indicates the mean, and thin traces are individual trials. Right: best fit model (pink) performance compared to behavioral data (black) for the block 1 to block 2 (blue to green) transition. Shaded areas in **c**,**d** and **g**–**i** indicate s.e.m.; *n* = 10 simulations at optimal parameters (25 parameter pairs tested) in **g**–**i**. a.u., arbitray units; Pr, probability.

**Extended Data Fig. 1:**
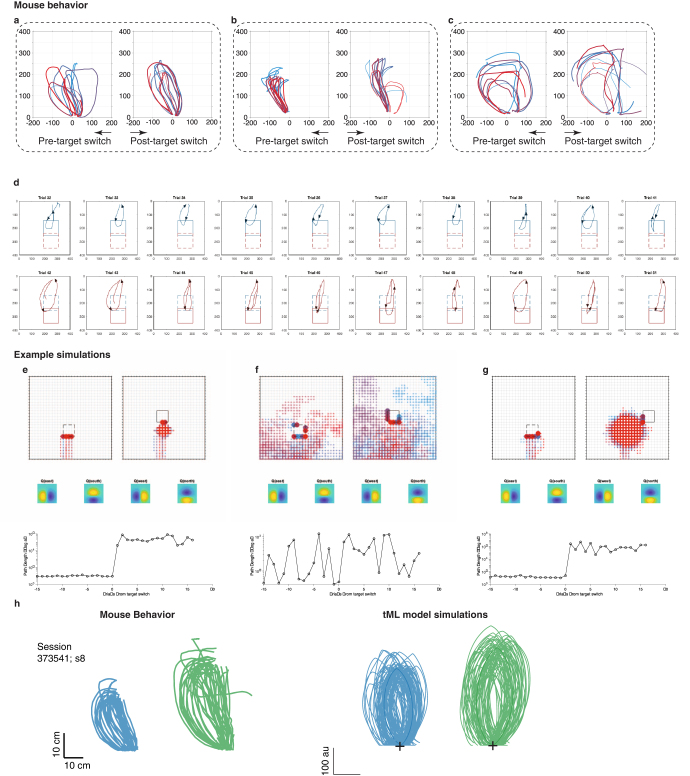
Exploration trajectories around target switch in example mouse behavior and version of optimal Q agents. **a-c**, Example data from 3 different mice illustrating a set of 10 trajectories before and after target switch. Thick lines indicate rewarded attempts. **d**, 10 rewarded trial trajectories around a switch in the target location (cyan, before; red, after). Illustrate the smooth scaling of trajectory amplitude after switch. One can also see the ‘stub’ of a return trajectory after a failed (unrewarded) run after the switch and then an immediate increase in trajectory amplitude in response. **e-h**, Simulations of an epsilon-greedy RL agent model exploiting an optimal action-value representation (that is product of Q learning) in the STF task. Upper panels show 20 simulated colored trajectories to target under optimal policy (policy shown in small heatmaps below). Lower graphs are the number of discrete steps (actions) taken to reach the target plotted as the log action count (path length). **e**, epsilon set to a typical value in the field of 0.05 exhibiting perseverance around the prior target on initial trials after a switch that leads to a consistent, large increase in path length. **f**. for comparison a random agent is shown (epsilon=1; policy irrelevant but shown for consistency). **g**, an optimal agent is shown exhibiting extremely long paths and perseveration around prior target when target is moved to a more distal location. Epsilon increased to 0.4 because agents with too low epsilon never find the new target on first attempts. *Note: even in e with standard target locations epsilon* = *0* + *optimal policy on target 1 yields agents that fail to ever discover new target* (*See* Methods*)*. Simulations are not full STF task (agent not required to return to reward location). Since most innovations in RL are related to more rapid and accurate convergence to optimal policies in large state spaces, we are effectively simulating consequences for exploration around a sudden change in target location in optimal models (in this sense). **h**, Comparison of a simulated session from model and an example session of trajectories to the near and far target locations.

To understand how mice shifted their foraging trajectories to intercept more eccentric targets after a change in location, we extracted individual foraging runs made by the animal immediately after a target transition. In individual sessions and across the data in aggregate, mice smoothly shifted their trajectories to take longer paths that were more likely to intercept the new target location (Fig. 2a–d). We found little evidence for a sudden jump in trajectory length as would be expected from undirected (biased random walk) search (Extended Data Fig. 1). The gradual change in trajectory amplitude, fluctuations even after tens of trials and absence of a large, discrete switch after transition (Fig. 2a–d) argues against the possibility that mice were switching between two fixed trajectories/policies. Rather, these data suggest that updates to the foraging trajectory depend upon recent experience evaluating outcomes of a trajectory.

The challenge in designing RL models of spatial navigation that match animal learning stems from two key issues. First, if the space of possible target locations is large, then RL agents can be slow to update due to the ‘curse of dimensionality’^36^. An agent needs to sample all^36^ or a sufficient number^31^ of discrete locations (states) to optimize its behavioral policy—a direct trajectory to the target. This limitation has typically been addressed using methods that generalize across state space even without sampling all states—from relatively local generalization among neighboring states, such as the ‘successor representation’^31^, to the use of deep learning to approximate functions that span high-dimensional state space^37^. Alternatively, distinct representations of the task can be considered^13^. Second, RL methods typically use a stochastic behavioral policy at each moment to explore. Approaches that add structure to an agent’s exploration of environmental state space can lead to substantial performance improvements^38^.

Although these refinements improve performance of RL models, there are still intriguing differences from observed navigational behavior in animals^39^. For example, the highly structured trajectories in the STF task both before and after a target switch (Figs. 1 and 2a–d and Extended Data Fig. 1a–d) are dissimilar from trajectories expected from agents with stochastic exploration policies and perseverative exploration of the prior target location (Extended Data Fig. 1e–g). This prompted us to consider alternative model representations that plausibly model animal agents but address these key challenges and more closely match observed behavioral learning after a target switch.

What might account for smooth changes in trajectory after a shift in target location? We previously described an RL formulation that exhibits smooth, continuous changes in forelimb trajectory amplitude consistent with behavioral learning in the NTF task (‘mean shift plus homeostasis’ (MeSH))^35^. Rather than using random exploration of trajectory kinematics, MeSH searches a continuous, low-dimensional space of trajectory amplitude. This approach successfully explained the relatively rapid time course of learning during closed-loop optogenetic feedback^35^, the bidirectional learning in a task very similar to the NTF task here^40^, and was consistent with a plausible biological implementation^35^. Nonetheless, it was unclear whether or how this approach could be modified to model foraging trajectories, especially navigational trajectories.

Here we describe a simple RL agent with a behavior representation and learning rule that can more closely match key features of observed foraging trajectories and their reward-dependent adaptations to a target switch in both task contexts (trajectory MeSH learning, ‘tML’ for simplicity; Fig. 2e and Methods). The tML agent uses recent reward experience to update two continuous parameters (speed and heading angle) governing the generation of dynamics that implement trajectories (Fig. 2e,f). A tML agent learned to smoothly change foraging trajectory amplitude to adapt to a new target location in tens of trials (Fig. 2g) analogous to our behavioral observations (Fig. 2c,d). Optimizing a forward learning rate and a trajectory variance parameter was capable of producing very good agreement with behavioral data in both STF (Fig. 2h) and NTF (Fig. 2i) tasks, indicating that a common algorithmic description could be sufficient to capture behavior as mice adapted trajectories around a target location switch (Extended Data Fig. 1h).

### Common hippocampal activity patterns independent of context

**Fig. 3:**
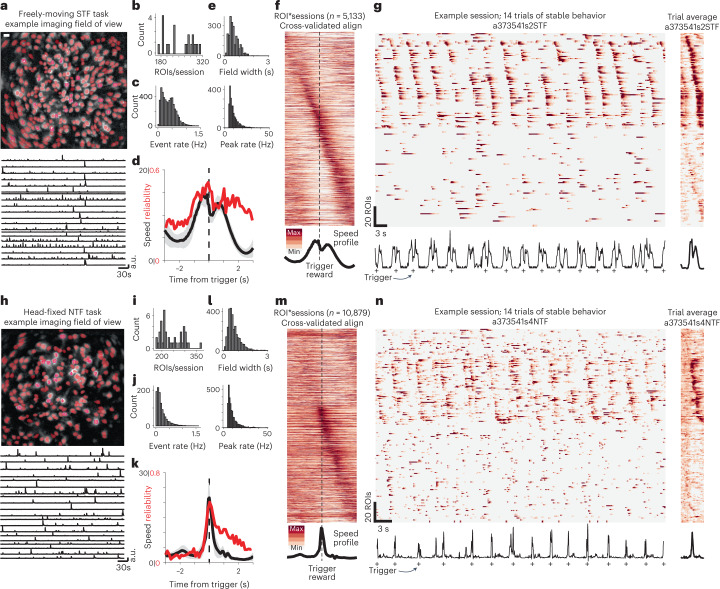
Sequential activity of dCA1 neural ensembles in navigational and non-navigational contexts. **a**,**h**, Example imaging field of view (standard deviation projection of Δ*F*/*F* image) for STF (**a**) and NTF (**h**) tasks with overlaid ROIs in red. Activity traces of several example ROIs are shown below. **b**,**i**, Number of ROIs recorded per session in STF (**b**) and NTF (**i**) tasks. **c**,**j**, Mean detected calcium event rate (left) and maximum inferred firing rates (right; for spike inference details, see Methods) per ROI in STF (**c**) and NTF (*j*) tasks. **d**,**k**, Mean (± s.e.m.) movement speed (black) and mean reliability of calcium activation profile (red) in STF (**d**) and NTF (**k**) tasks. **e**,**l**, Halfwidth of suprathreshold calcium events within trials in STF (**e**) and NTF (**l**) tasks. **f**,**m**, Sequential alignment of normalized calcium profiles for a held-out half of trials for all ROIs in all sessions imaged in STF (**f**) and NTF (**m**) tasks. Mean speed trajectories as in **d**,**k** plotted below for comparison. **g**,**n**, Imaging data for a run of 14 trials during performance of STF task (**g**) and NTF task (**n**). ROIs were sorted by first sorting ROIs above and below median activation and then within those groups sorting by latency of peak activation from movement onset. Average of example data for all trials within the session is shown at right. Lower traces show corresponding movement speed traces, and successful reward triggers are indicated by crosses.

**Extended Data Fig. 2:**
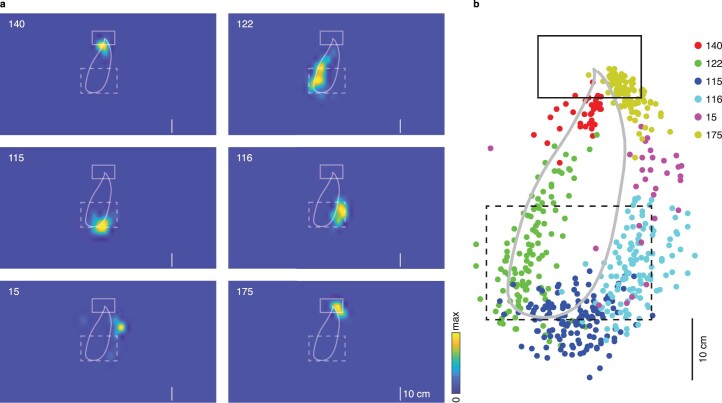
Example place fields distributed along foraging trajectories. **a**, Place fields of 6 example neurons overlaid on the average foraging trajectory in an example STF session. Dashed rectangle: target area; Solid rectangle: collection area; Closed circle: average of foraging trajectories; Top left number: ROI id number. **b**, Locations of calcium spikes from the example neurons in the same session as (a). Each dot marks the location of one calcium spike from a neuron. These neurons were sequentially activated along the foraging trajectories. Top right: neuron identity pseudocolor legend.

**Extended Data Fig. 3:**
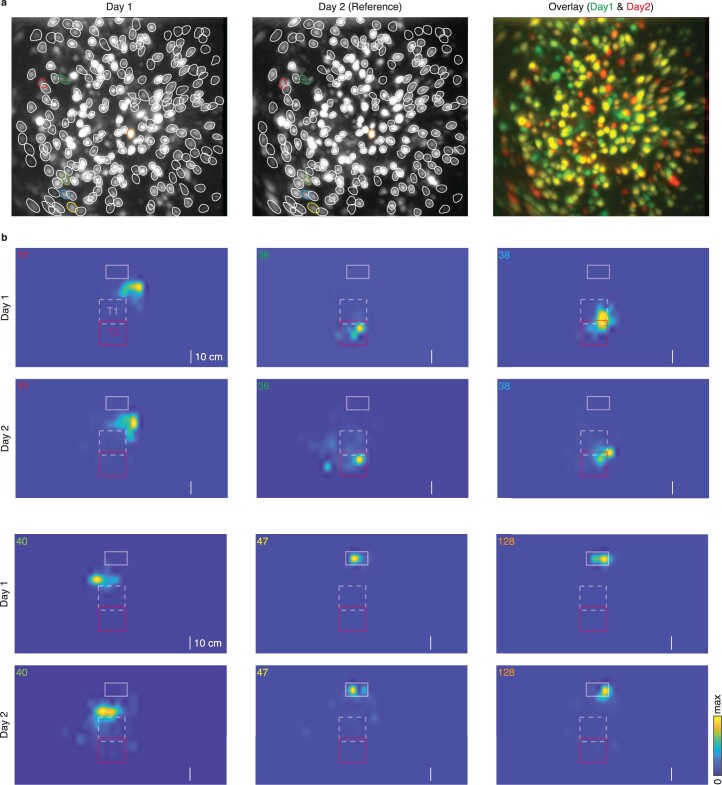
Stable imaging of individual dCA1 neurons across days. **a**, Example response images (maximum intensity projection - average intensity projection) of the same field of view across days. Left: aligned response image on Day 1 overlaid with the ROIs detected on both days; Middle: reference response image on Day 2 overlaid with ROIs detected on both days; Right: pseudocolor overlay of the response images on both days. **b**, Stable place fields of 6 example neurons across days. Dashed white rectangle: target area1; Solid red rectangle: target area2; Solid white rectangle: collection area; Top left number: neuron identity with the same color as that of its ROI in (a).

We next examined neural activity during performance of these tasks using epifluorescence imaging of dCA1 principal neuron activity with genetically encoded calcium indicators (GCamp6f) and a head-mounted miniscope^41,42^ (Methods). Many dCA1 neurons exhibit clear place fields within circumscribed regions of the environment. When an animal navigates along a reliable trajectory, this leads to a sequential activation of place cells^6,43^ that depends upon the spatial context^44^. We observed dCA1 place field activity distributed along the foraging trajectories (Extended Data Fig. 2). The reliable trajectories taken in the STF task (Fig. 1) resulted in reliable, sequentially ordered activity across a population of dCA1 principal neurons (*n* = 5,133 regions of interest (ROIs) × sessions; Fig. 3a–e) as revealed in an alignment of peri-movement time histograms (PMTHs) of activity (Fig. 3f) and apparent in individual trials (Fig. 3g). Activity of a subset of individual dCA1 neurons could be stable across days (Extended Data Fig. 3).

We observed a number of analogous properties in CA1 ensemble activity during the STF and NTF tasks. For example, in the head-fixed NTF task, the activity of dCA1 cells exhibited a similar heavy-tailed distribution^4,5^, although the calcium event rate was reduced (Fig. 3c,j; *P* ≪ 0.001, rank-sum test). The duration and peak rates of active cells were very similar (Fig. 3c,j,e,l). We next examined the responses of individual dCA1 neurons aligned to when the forelimb movement triggered the water reward. Alignment revealed robust time-locked responses in a subset of cells and sequential activation of hippocampal neuronal ensemble (Fig. 3m). Similarly to the freely moving STF task, sequential activity was robustly observed in single-trial activity during task performance (Fig. 3g,n).

**Extended Data Fig. 4:**
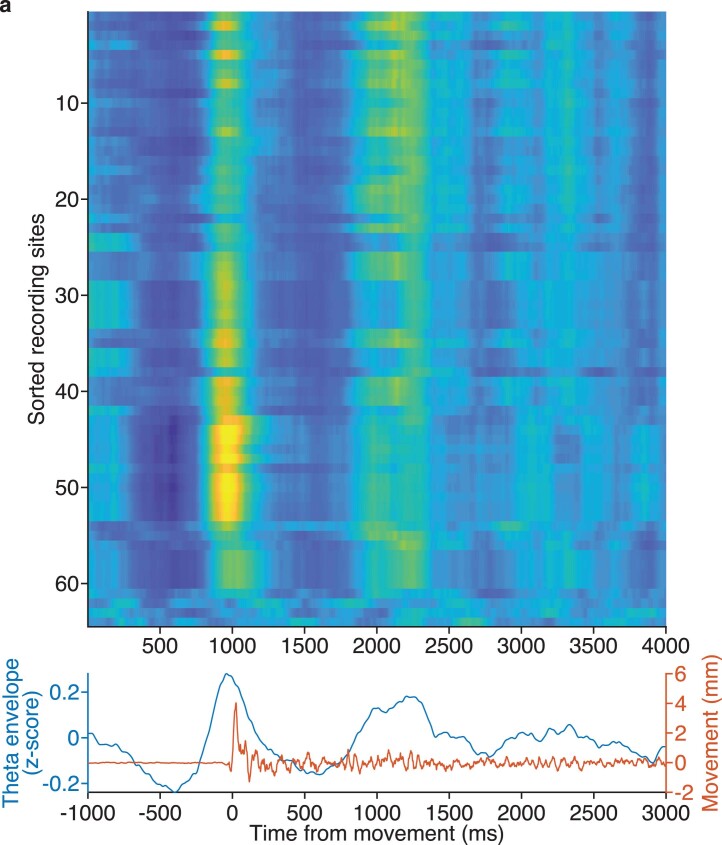
Forebrain activity was modulated at theta frequency in NTF task. Data from a 64-channel probe recording from dorsal striatum in the joystick task from previously published data^32^. The mean amplitude envelope of filtered LFP signal (bandwidth: 4-12 Hz) is plotted for all 64 channels averaged across all joystick movements. Lower plots show the mean amplitude envelope change across all trials; all channels (cyan) compared to the mean joystick movement (red; sampled at 1 kHz). Note: second peak in theta envelope is around the time of mean reward delivery (1 second after threshold crossing).

To evaluate whether the trial-by-trial consistency of individual dCA1 neuron responses was related to movement, we calculated the reliability of a response (Methods) as a function of the instantaneous velocity of the body (freely moving STF task; Fig. 3d) or the limb (head-fixed NTF task; Fig. 3k). In both cases. we found that the reliability of dCA1 responses was significantly correlated with movement (STF task: Fig. 3d, Pearson’s *r* = 0.21, *P* ≪ 0.001; NTF task: Fig. 3k, Pearson’s *r* = 0.41, *P* ≪ 0.001). Reliability of activity was lowest during the inter-trial interval (ITI) before movement onset (Fig. 3d,k) but elevated throughout the active behavior of a trial (joystick/navigational movement through anticipatory licking and reward consumption) in both task contexts (Fig. 3d,k). Although not possible to resolve in calcium imaging due to low temporal resolution, theta frequency modulation of forebrain local field potential was associated with performance of the NTF task in previously reported dorsal striatal recordings^33^ (Extended Data Fig. 4) and has been associated with related non-navigational behavior in hippocampus^45^. This suggests that forelimb movements reflect an active state distributed across multiple forebrain circuits^46^, perhaps analogous to freely moving navigation^47^.

### Trajectory decoding depends upon spatial context

As noted above, place fields in dCA1 activity are apparent as sequential patterns of activity observed on individual trials in both tasks (Fig. 3g,n). There is substantial evidence that, in navigational foraging behaviors, dCA1 place cells tile the environment and, thereby, allow for an accurate encoding of allocentric position along a trajectory and the potential to represent target and reward locations^30^. A similar argument might be made for joystick trajectories; however, it is less clear whether the sequential patterns of dCA1 activity observed during head-fixed forelimb movements encode joystick position.

**Fig. 4:**
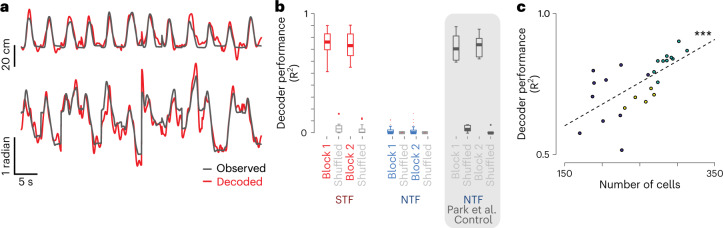
Decoding of behavior trajectory with dCA1 ensemble activity. **a**, Example traces showing amplitude (upper) and angle (lower) of concatenated foraging runs (black traces) and the performance of a linear ensemble decoder trained on simultaneous dCA1 activity for a single session of STF task (red traces). **b**, Summary data showing consensus decoder (Methods) performance (coefficient of determination, R^2^) in the STF (red, left) and NTF (cyan, right) tasks compared against a shuffled (permuted decoder weights) control (gray). A positive control for decoding was estimated from data in ref. ^48^ recorded in the motor cortex during performance of an NTF task variant. Data were downsampled to imaging rates, and decoding performance was assessed (gray shaded box; see Methods for details). Two-tailed rank-sum tests confirmed significant differences between data and shuffled controls as well as between the NTF and STF groups (STF: *n* = 25 sessions and *N* = 3 mice; NTF: *n* = 26 sessions and *N* = 5 mice) (*P* ≪ 0.01). **c**, Population imaging decoder performance in the STF task is plotted as a function of the number of ROIs imaged in a given session. Dashed line reflects the best-fit linear approximation reflecting a significant Pearson’s correlation (*P* < 0.001). Box plot properties: center: median; edges: 25th–75th percentiles; whiskers: extrema; outliers plotted individually.

To address this question, we trained continuous-time linear decoders to examine whether trajectories could be reliably decoded in both task contexts (Methods). We found that navigational foraging trajectories could be very accurately decoded from dCA1 activity inferred from calcium imaging as expected (Fig. 4) and exemplified in activation of neurons with place fields along the trajectory (Extended Data Fig. 2). In contrast, there was little ability to decode forelimb trajectories using inferred spiking activity in dCA1 (Fig. 4b). One concern is that the shorter duration of forelimb movements might render decoding impossible. As a positive control, we used an electrophysiological dataset recorded from the primary motor cortex in the same task and shown to provide excellent decoding^48^. We downsampled and smoothed the electrophysiological data to approximate inferred spike rates in calcium imaging and found that decoding performance was largely maintained (Fig. 4b).

### dCA1 synchronous population activity remaps across contexts

Previous RL models of navigational tasks invoked a computation in which a given location is associated with reward receipt. Place fields and specialized reward responses are a critical neural representation for such associations^30^. The tML model postulates a distinct representation in which the obtained reward is used to update a generative parameter governing future trajectories—a computation that could be well-suited to updates during an SWR-like event. The probability of observing SWR events is correlated with active recall of remembered (non-navigational) items and retrospective replay of experience in non-navigational contexts in human subjects^19,24^; however, SPEs in a non-navigational context have yet to be described in mice. Moreover, in navigational tasks, SWR events are proposed to be a mechanism for prospective planning of future navigational trajectories^49^ and retrospective replay of recent experience^14,21^; however, the extent to which SPEs might also encode egocentric information about navigational trajectories (as expected in the tML model) remains unclear ^44^.

**Fig. 5:**
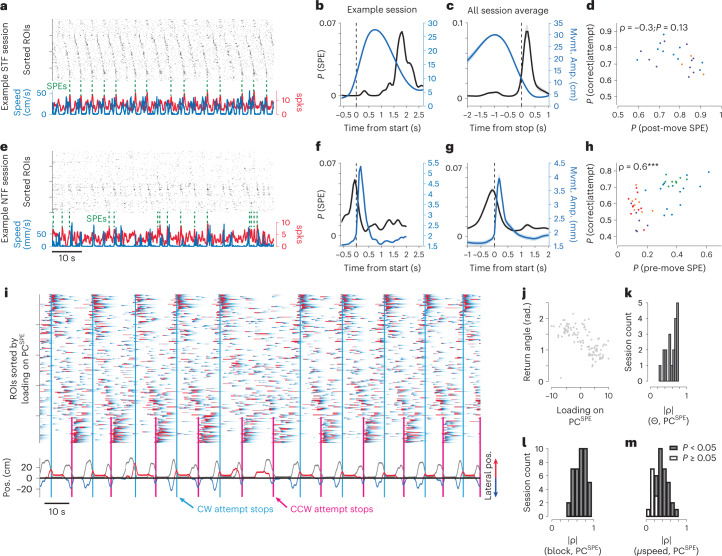
Distinct timing of SPEs in navigational and non-navigational contexts. **a**, Example data from a session of STF task. Raster plot shows inferred spikes from sorted ROIs during foraging trajectories (Methods). Lower traces: running speed (blue) overlaid with summed spike counts across the population (red). Vertical dashed lines indicate detected SPEs. **b**, Average position aligned to trajectory start (blue) is overlaid with the probability of observing an SPE event (black) for an example session. **c**, For all mice (3) and sessions (25), the mean amplitude (distance from reward port, blue) and probability of observing an SPE (black) are plotted aligned to trajectory stop. **d**, Fraction of successful trials across the entire session plotted against max P (SPE) per trial for all sessions of STF task. Colors indicate individual animals. **e**, Example data from a session of NTF task as in **a**. **f**, The average joystick position aligned to movement start (blue) is overlaid with the probability of observing an SPE event (black) for an example session. **g**, For all mice (5) and sessions (26), the mean amplitude (dislocation of joystick from the origin, blue) and probability of observing an SPE (black) aligned to start of trial-initiating forelimb movement. Shaded areas in **c** and **g** indicate s.e.m. **h**, Analogous plot as **d** but for NTF task. **i**, Δ*F*/*F* transients are plotted for an example session and sorted according to loading onto the principal component that best captured ensemble activity during SPEs (PC^SPE^). Lower traces: the position relative to the reward port (gray) overlaid with color-coded lateral position (blue: negative; red: positive) from the water port. Distinct SPE populations were observed around the end of clockwise (CW; cyan) and counterclockwise (CCW; magenta) trajectories. **j**, For an example session, the return angle is plotted as a function of loading on PC^SPE^. **k**, Pearson correlation coefficients between return angle (theta) and loading onto PC^SPE^ for all sessions. **l**,**m**, For the NTF task, correlations between target block (**l**) or mean trajectory speed (Methods; **m**) and loading on PC^SPE^ for all sessions. Shaded bars in **k**–**m** indicate statistical significance.

**Extended Data Fig. 5:**
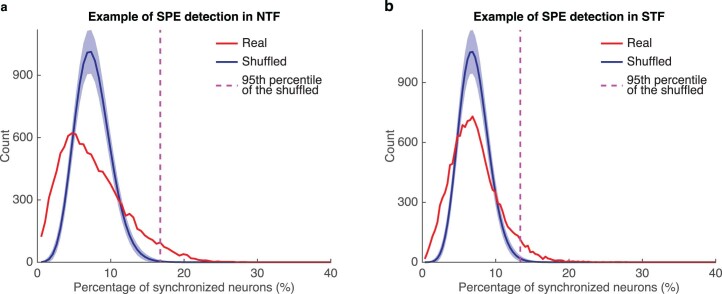
Detection of SPE events. **a**, Histograms of synchronized neurons in an example NTF session and in its corresponding shuffled datasets. The shuffled datasets were generated by shuffling the spike-timing but keeping the interspike intervals for each neuron and repeating the shuffling procedure 1000 times. Shaded blue line: mean ± s.e.m of the 1000 shuffled datasets; Magenta dashed line: statistical threshold for SPEs detection. Any imaging frame with more synchronized neurons than the threshold was detected as a SPE (Fig. 5e). **b**, same as (a) but for an example of STF session (Fig. 5a).

Several lines of evidence suggest that SPEs observed with calcium imaging correspond to SWR-associated population events observed in electrophysiological recordings^50,51^. Here we define SPEs as near-simultaneous activation of a significant fraction of the imaged population (Extended Data Fig. 5; roughly 15% of the imaged population within ~200 ms). Using this conservative detection approach, we observed SPEs at a rate of roughly 0.5 Hz throughout all of our imaging datasets (Fig. 5a,e), consistent with previous observations using imaging^50,51^ or inferred SWR-associated events^19^. In both tasks, and analogous to previous observations with imaging^51^, we observed multiple clusters of dCA1 ensembles active during SPEs. On average, we observed similar numbers of SPE clusters per session (silhouette method): 3.6 ± 0.98 and 3.7 ± 1.92 (s.d.) in the STF and NTF tasks, respectively.

SPEs occurred largely in the absence of movement; however, there was a clear difference in the timing of SPEs across spatial contexts even in the same mouse trained on both STF and NTF tasks. In the STF foraging task, we observed SPEs primarily at the termination of a foraging run as the mouse returned to the reward collection area (Fig. 5a–c), similarly to previous reports with electrophysiological recordings^14^. In contrast, in the same mouse during a non-navigational NTF task, SPEs were primarily observed just before initiation of a trial (Fig. 5e–g). The probability of observing SPEs before trial initiation was strongly correlated with the quality of task performance in the non-navigational task (Fig. 5h; ⍴ = 0.6 and *P* ≪ 0.001). In the navigational STF task, the probability of observing an SPE was enhanced on correct foraging runs that yielded reward as compared to incorrect attempts (0.77 ± 0.18 versus 0.49 ± 0.08 (s.d.); *P* ≪ 0.001, rank-sum test) but was not significantly correlated with overall performance accuracy (Fig. 5d; ⍴ = −0.3 and *P* = 0.13).

**Extended Data Fig. 6:**
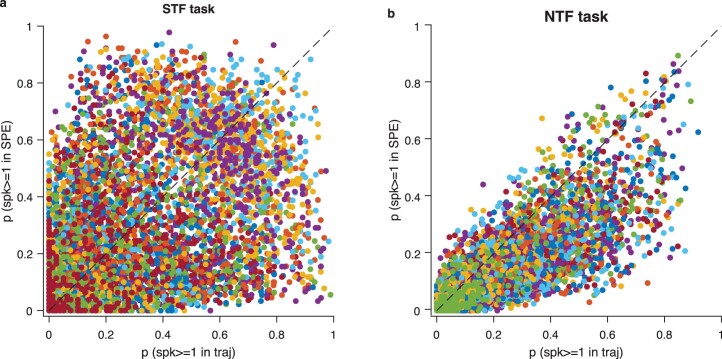
Comparison of activity of individual neurons along foraging trajectory and those in SPEs for STF task (a) and for NTF task (b). Each dot represents one ROI. Inferred spike times (see Methods) were used to compute the probability of observing a spike either during the trajectory (x-axis) or in the window of time of elevated SPEs (y-axis; see Fig. 5 for elevated SPE probability windows). Color codes reflect different sessions / mice from which individual spike probabilities were obtained.

We next asked whether SPE ensembles could provide information about the scaling of navigational trajectories in the STF task. Neuronal ensembles active in SPEs tended to be recruited during execution of navigational and non-navigational forelimb trajectories (Extended Data Fig. 6); however, there was substantial variance in this relationship, and SPE ensembles could also contain additional information about the trajectory critical for learning (such as in ‘non-replay’ events^52^). Moreover, in the NTF task, ensembles recruited during the trajectory were insufficient to decode kinematics, suggesting that additional information in SPE ensembles might be critical. To capture the variance of individual SPEs, we projected the population vector onto its first principal component (PC^SPE^). First, by sorting imaging data according to loading weight on this PC^SPE^, one can observe distinct patterns of active cells during SPEs at the end of foraging trajectories. For example, classifying trajectories into those that were counterclockwise and clockwise reveals distinct ensemble clusters associated with distinct trajectory headings (Fig. 5i). All imaging sessions had a significant correlation between PC^SPE^ and return trajectory heading (⍴^2^: 0.38 ± 0.17 (s.d.); *P*: 3 × 10^−3^ ± 6.8 × 10^−4^), indicating that SPE ensembles in the STF task encode parameters of recently taken trajectories (Fig. 5j,k).

**Extended Data Fig. 7:**
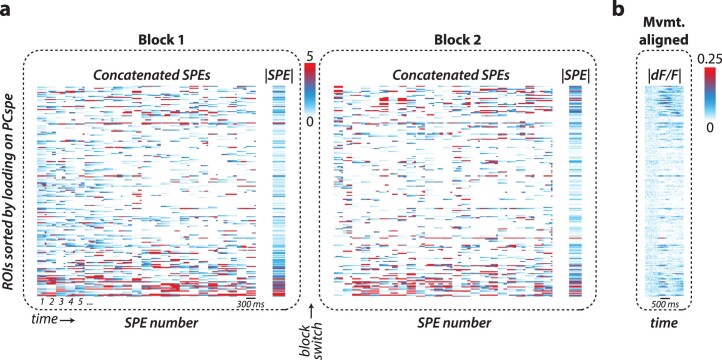
Example session data for SPE encoding of NTF task blocks. **a**, A matched number of SPE events were retrieved from each Block of the NTF task and concatenated into a single matrix. The indexing of ROIs along the y-axis is sorted by the loading weight onto the PC^SPE^ (see Fig. 5 and Methods). At right in each grouping is shown the mean SPE (|SPE|) responses of individual ROIs for the block. Color indicates dF/F activity as reflected in the colorbar. **b**, The mean perimovement time histogram (as in Fig. 3) for this session and with the same ROIs order as in (a).

For a forelimb movement, the amplitude of the movement is also thought to be represented before and at initiation of movement execution at least in cortico-basal ganglia circuits. This suggests that the hippocampal SPE activity before movement might also encode aspects of the upcoming movement. To assess this possibility, we considered the potential encoding of the current target block and an estimate of the current speed scaling parameter—a parameter of an inferred behavioral policy distinct from kinematics of the current movement. In both cases, we observed significant correlations in all mice (*N* = 5) and in 100% and 76% of individual sessions, respectively (Fig. 5l,m and Extended Data Fig. 7).

### Effects of dCA1 perturbation depend upon context

Replay of hippocampal activity has been proposed to play a critical role in updating representations of value in non-navigational^19^ and spatial, navigational tasks^26^. Most computational work has argued that hippocampal activity represents environment state (location, time, stimulus identity, etc.^30^), which is then associated with inferred value. SWR-associated replay events are then argued to provide efficient generalization across states and/or planning future trajectories^26,31,49^. Although, it should also be noted that most SWR-associated events may not be replay of spatial trajectories^49^, and ‘non-replay’ events contain information that may also be as or more critical for learning new goals^52^. The tML model exploits a lower-dimensional representation of non-navigational and navigational actions and, thus, invokes a distinct (additional) computation in which a foraging trajectory is generated from parameters that are updated dependent upon reward receipt. Consistent with this model, we found that SPE events in both STF and NTF task contexts encoded information about the amplitude and heading of recently completed or intended foraging trajectories and were correlated with reward receipt (STF; Fig. 5a–c) or task performance (NTF; Fig. 5h) across spatial contexts, respectively. Thus, we next asked whether there is causal evidence that dCA1 activity at the time of SPEs was critical for learned updates to foraging trajectories.

**Fig. 6:**
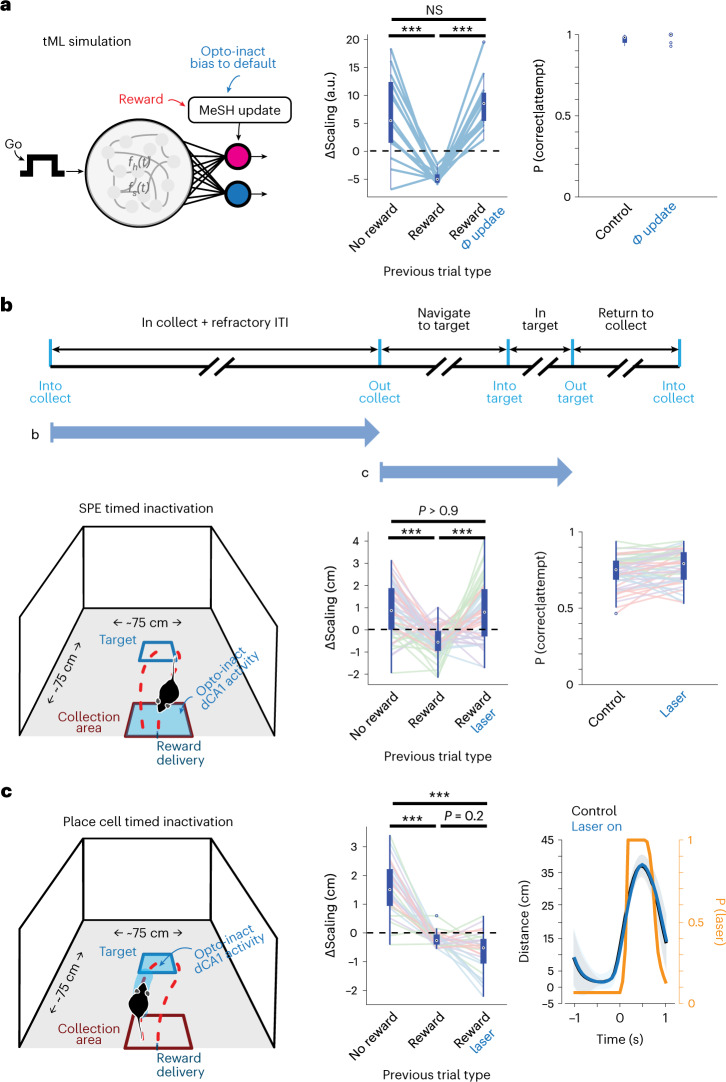
Optogenetic inactivation of SPE-timed activity in dCA1 impairs trajectory updating in STF task. **a**, tML model (Fig. 2) is extended to simulate optogenetic perturbations in STF task (Methods). Multiple perturbations were examined (Table 1 and Extended Data Fig. 8d) with a specific instance—inactivation causes reversion toward the default policy—shown. Middle panel shows a difference in observed trajectory amplitude scaling depending upon whether the previous trial was unrewarded (left), rewarded (middle) or rewarded + impaired update (right). Right panel shows performance for control and impaired update (*n* = 15 simulations). **b**, Top: schematic of optogenetic perturbations during different phases of STF task. Bottom left: Optogenetic inactivation was delivered on catch trials (≤30% of trials) in closed-loop between entry (reward consumption) and exit to the collection area (when SPEs were observed; Methods). Bottom middle: the change in trajectory amplitude (ΔScaling) conditioned upon whether the previous trial was unrewarded (left), rewarded (middle) or rewarded + laser (right) for all mice and all sessions (*n* = 4 mice an *N* = 13 sessions per mouse). Bottom right: Probability that a given attempt was rewarded (P(correct|attempt)) is plotted conditioned upon whether the previous trial was inhibited by laser activation. Colors indicate different animals. **c**, Left: schematic of STF task and scheme for optogenetic inactivation experiments during navigational trajectories. Inactivation was delivered only on catch trials (≤30% of trials) in closed-loop after exiting the collection area and ceased upon exit from peri-target area (when place cell activity, but not SPEs, were observed). Middle: same as **b** (*n* = 4 mice and *N* = 7 sessions per mouse). Direct comparison of laser trials across conditions was also significant (Kruskal–Wallis test, *P* < 0.01). Right: Trajectories summarized by mean distance from port aligned to trajectory start are shown for control (black) and inactivated (blue) trials; shaded area indicates s.e.m. The average timing of laser activation is shown in yellow on the right axis. There was no effect on trajectories during laser activation. ***P < 0.001, Kruskal–Wallis test with multiple comparison corrections. Box plot properties: center: median; edges: 25th–75th percentiles; whiskers: extrema; outliers plotted individually. NS, not significant.

**Extended Data Fig. 8:**
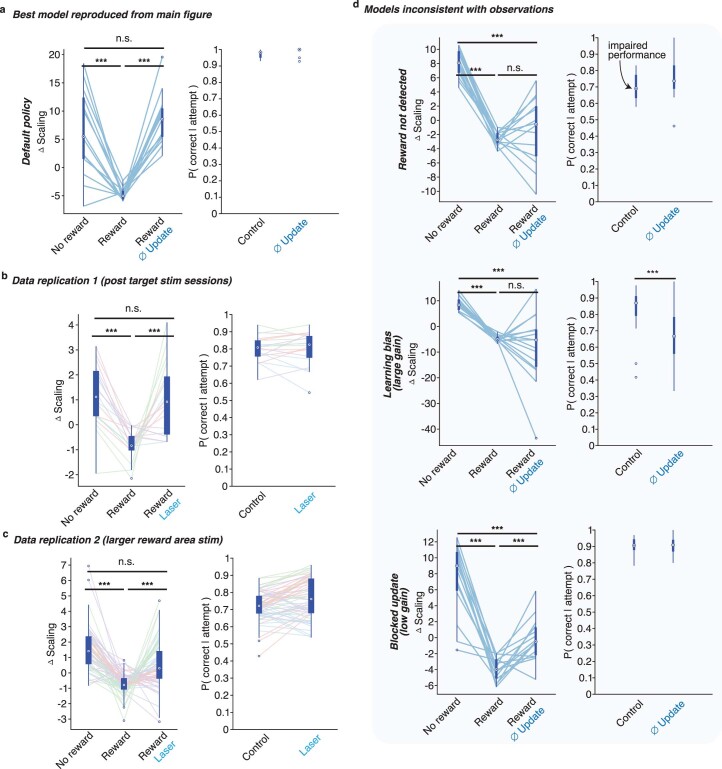
Additional data and model simulations for STF task optogenetic perturbations. **a**, Replotting of simulation data shown in Fig. 6a for comparison (n = 15 simulations). **b**, Plotting data just for a subset of sessions which followed the target-inactivation sessions (Fig. 6c) consistent with data shown in the main figure (n = 4 mice, N = 5 sessions/mouse). **c**, A further experiment was done using a larger reward collection area spatial trigger for inactivation (n = 4 mice, N = 16 sessions/mouse). Analogous results were obtained. **d**, A collection of distinct simulation results as described in the main text and Table 1 (n = 15 simulations). None of these simulations exhibited a fully consistent pattern of effects with experimental results (b-c). ***, p < 0.001 Kruskal-Wallis test with multiple comparison corrections; Box plot properties: centre: median; edges: 25th-75th %, whiskers: extrema, outliers plotted individually.

We first consider the predicted changes in behavior using simulations of impaired updating in the tML model in the context of the STF task (Table 1). An important feature of the tML model is that, even during stable performance with a fixed target, reward receipt still actively stabilizes parameters^35^. This can be seen by comparing the change in trajectory parameters conditioned on whether the previous trial was rewarded or unrewarded (Fig. 6a; ‘no reward’ versus ‘reward’) even during stable behavioral performance (Fig. 6a; P(correct|attempt)). Given the absence of a tight correlation between STF task performance and SPE probability, one would expect no deficit in performance from optogenetic manipulations. However, if place cell sequences were critical for ongoing navigation, a deficit in performance or altered trajectories might be expected during acute inactivation. To assess the predicted behavioral consequences of dCA1 inactivation, we considered several possible implementations of impaired updating to generate a family of predictions inspired by distinct interpretations of neural correlates (Table 1 and Extended Data Fig. 8d).

**Table 1:** Summary of tML simulations of optogenetic perturbation effects on behavior

Simulation name	Adjusted parameters	Logic of the adjustment	Results in figure panels
**Default policy**	*µ*_*k+1*_ = *µ*_*1*_	Observation: Trajectory encoding in SPE ensemble and reward-dependent SPE occurrence and inactivation during behavior has no effect on executed trajectories (indicating a putative additional extra-CA1 control of navigation).	Fig. 6 and Extended Data Fig. 8a–c
		Interpretation: No CA1-dependent updating of trajectory and behavior reverts back to a default (possibly extra-hippocampal) policy.	
**Reward not detected**	*target_hit* =*=* *0*	Observation: reward-dependent SPE occurrence.	Extended Data Fig. 8d
		Interpretation: Animal needs the SPE in CA1 to detect presence of reward.	
**Learning bias (large gain)**	*µ*_*k+1*_ = *µ*_*k*_ + 10*∂µ	Observation: trajectory encoding in SPE ensemble and reward-dependent SPE occurrence.	Extended Data Fig. 8d
		Interpretation: Inhibition of CA1 SPE leads to gross misrepresentation of prior trajectory and, thus, an exaggerated MeSH (error-based) update.	
**Reduced learning (low or 0 gain)**	*µ*_*k+1*_ = *µ*_*k*_ + [0,0.1]*∂µ	Observation: trajectory encoding in SPE ensemble and reward-dependent SPE occurrence.	Extended Data Fig. 8d
		Interpretation: Inhibition of CA1 SPE leads to inaccurate estimate of error and, thus, a reduced or non-existent MeSH update.	

To assess potential roles of SPE-timed dCA1 inactivation, we considered several possible implementations of impaired updating summarized in the table. Parameters of MeSH update manipulated (see Methods and codebase) used to generate predictions are shown as well as connection to experimental data and proposed interpretation.

To examine the contributions of dCA1 activity at specific phases of task performance required the precise control of optogenetics. We took advantage of a mouse line that expresses the optogenetic activator channelrhodopsin-2 (ChR2) in inhibitory neurons (using VGAT-ChR2-EYFP mice; Methods) to suppress activity of principal cells in dCA1 at modest light intensities^53^. We first performed a pair of manipulations in the STF task using online detection of performance. First, we targeted the period when the mouse returned to the reward collection port—that is, when SPEs occurred (Fig. 6b). We compared this to the period of time around when the mouse intercepted the target location during active navigation (Fig. 6c)—that is, when place cell sequences occurred—but SPEs were not detected.

The experimental manipulations targeted to the time of SPEs were uniquely consistent with simulations in which inactivation was simulated as a reversion toward a default policy on subsequent trials (Fig. 6a,b, Table 1 and Extended Data Fig. 8). In contrast, dCA1 inactivation during active navigation around the target location did not produce any significant alteration in reward-dependent updating on a subsequent trial, nor did it produce a clear perturbation of ongoing movement during inactivation (Fig. 6c). These data thus provide causal evidence that SPEs in dCA1 at the time of reward collection are likely critical for updating of future trajectory parameters.

**Fig. 7:**
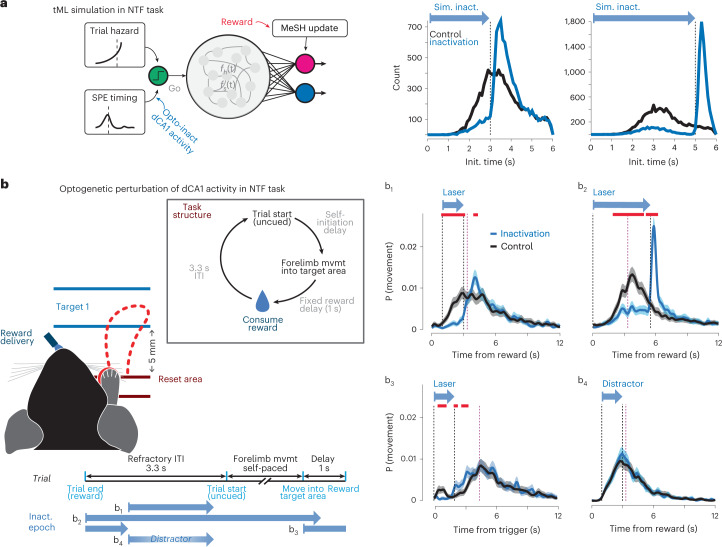
Optogenetic inactivation of SPE-timed activity in dCA1 impairs initiation of non-navigational forelimb movements in NTF task. **a**, Schematic of a modified version of the tML model incorporating a plausible, internal ‘go’ signal for trial initiation. Initiation is primarily determined by a standard assumption of an intertrial hazard function^70^ adapted to the average ITI and governing timing of self-initiation of a forelimb movement. In addition, we postulate that the occurrence of an SPE can increase the probability of initiation (see Methods for details). Using a continuous approximation to the empirical distribution of SPE onset times during the ITI, this model approximately recapitulates the distribution of initiation times in control conditions (black lines at middle and right). Laser inactivation was simulated as a strong reduction in SPE probability, with a spike in SPE probability around the offset of inactivation (consistent with previous observations in VGAT-ChR2 mice^58^). Expected timing of initiation during inactivation trials is plotted in blue (middle, brief inactivation; right, sustained inactivation). **b**, Schematic of the head-fixed NTF task used for optogenetic inactivation experiments (*N* = 3; also see replicate in Extended Data Fig. 9). Preparation was similar to Fig. 6b. On individual catch trials (≤30% of trials), inactivation was delivered in closed-loop 1 (b_1_) or 0 (b_2_) seconds after reward delivery (times at which SPEs were observed) or during reward collection (b_3_), or a visible blue light flash outside the brain was delivered as a distractor (b_4_). For each experimental condition, histograms of movement initiations per trial are shown for control (black) and inactivation (cyan) conditions. Black dashed lines indicate the beginning and end of blue light. Purple lines indicate end of ITI (uncued trial start; see Fig. 1 for task details). Shaded areas in b_1_–b_4_ are the mean movement rates, and 0.025–0.975 CIs from 1,000-fold shuffles. Red lines indicate times in which a significant difference between control and inactivation were observed (permutation test).

We observed distinct SPE timing in the freely moving STF task compared to the head-fixed NTF task. Previous work using lesions and pharmacological inactivation demonstrated key roles for dCA1 in non-navigational tasks^7,54,55^. The reliable timing of SPEs before trial initiation in the NTF task suggests that they could play a prospective role (planning or initiation) in forelimb trajectories (Fig. 5f,g). It has long been proposed that forelimb movement requires a ‘go’ signal specific for the timing of movement initiation^56^ and apparent in motor cortical activity^57^. Thus, we next simulated a version of the tML model in which movement initiation was a probabilistic function of an inferred internal hazard function governing action initiation and our measured statistics (timing and probability; Fig. 5g) of SPE events in behaving animals (Fig. 7a). This model implementation yielded predicted distributions of initiation latency very similar to those observed in control trials (Fig. 7a, middle and right). However, action initiation was systematically delayed as a function of the duration of blocked SPE events in simulation (Fig. 7a, middle and right). These simulations thus provide some further quantitative evidence that observed SPE statistics are consistent with a role in action initiation in the NTF task.

**Extended Data Fig. 9:**
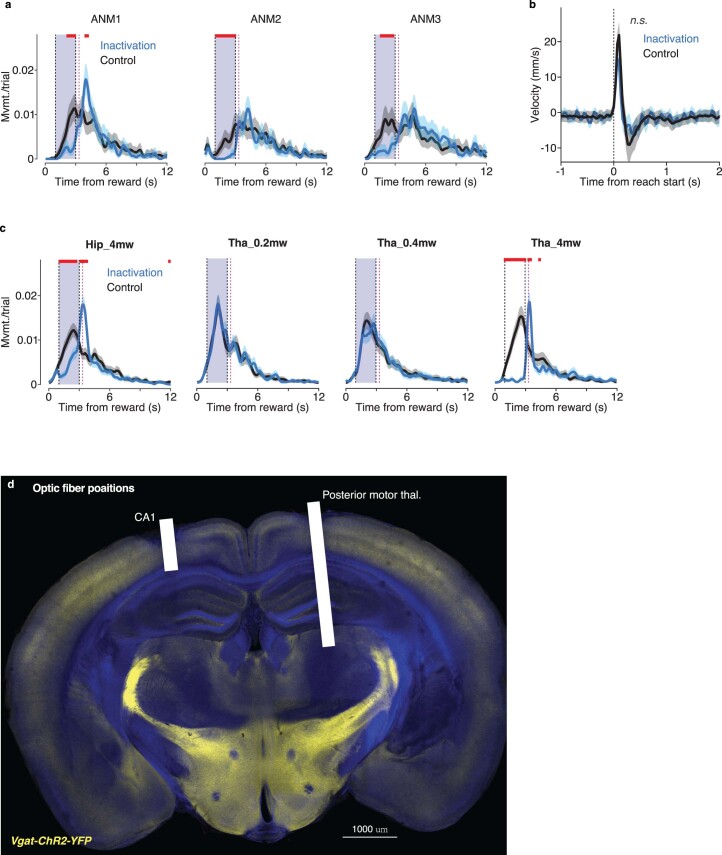
Optogenetic inactivation in the NTF task. **a**, Replication of results in Fig 7b1 in an additional 3 animals. **b**, Comparison of movement velocity during control and optogenetic inactivation of dCA1. **c**, Additional control experiments in which a fiber was implanted below hippocampus (~2.5 mm depth; 1 mm deeper than dCA1 fiber placement) to estimate potential effect of light spread to underlying motor thalamus areas. Up to 10% power to approximate light spread (note this is a simulation of a higher intensity than expected from diffuse light spread) fails to produce any effect on movement. Strong inactivation of motor thalamus is sufficient to completely block movement (unlike dCA1 inactivation which only biases against initiation). **d**, A thick slice was cleared and imaged on a light sheet microscope. Thick slices allowed reconstruction of hippocampal and thalamic targeting optical fibers to be well estimated. Scale bar: 1 mm. Nominal depth of optical fiber for thalamus is ~1.5 mm deeper than CA1 targeting fiber. Shaded line in **a-c**: mean and 0.025-0.975 confidence interval by 1000-fold shuffles. Two-sided permutation test in **a-c**; n.s.: no significance.

We next examined the effect of optogenetic inactivation of dCA1 (Fig. 7b) for two durations (brief, Fig. 7b1 and Extended Data Fig. 9a; and sustained, Fig. 7b2), similar to those used in tML simulations. On trials with optogenetic inactivation, mice substantially reduced the probability of initiating a joystick movement and biased toward initiation after the end of optogenetic illumination (Fig. 7b1–2). When movements were initiated—upon release of or, rarely, during inactivation—mice were still capable of making coordinated movements of the joystick to trigger reward (Extended Data Fig. 9b), indicating an absence of a block of movement per se similar to perturbation during navigation in the STF task (Fig. 6c) and distinct from inactivation of an obligate structure for movement^48,58^.

We performed a set of controls to assess how specific this optogenetic inactivation effect was to dCA1. For example, we considered whether the modest amount of illumination could alter initiation or become a cue; however, visual distractor flashes and inactivation during reward collection in control experiments did not affect trial initiation (Fig. 7b3–4). Previous work demonstrated that even weak illumination that penetrates below the hippocampus can produce modest changes in activity in the underlying thalamus^59^. Although motor thalamic nuclei are located >2 mm below our fiber tips where blue light power is substantially reduced^58^, we performed an additional control experiment. We implanted fibers below the hippocampus and directly applied ~10% peak power to control for partial effects due to light spread. We found that this illumination, replicating potential light spread although at a substantially higher intensity than predicted from scattering^60^, produced no effect on movement initiation in the NTF task context (Extended Data Fig. 9c,d). At the same time, high-intensity illumination of motor thalamic nuclei can produce a profound and complete suppression of forelimb movements, as described previously for reach-to-grasp movements^61^.

## Discussion

Many lines of evidence indicate that the CA1 region of the hippocampus plays a critical role in RL in both navigational^12,30^ and non-navigational^13^ contexts. Although it is generally agreed that CA1 is a critical component of a cognitive map of the environment, there are diverse proposals for the specific role of hippocampal representations in spatial memory, social behavior, foraging, decision- making and/or RL^15,17,25,26,31,49,62^. One critical outstanding question is whether a putative cognitive map in the hippocampus represents the relational structure of the environment independent of the spatial/behavioral context. We studied two foraging tasks that are highly similar in their relational structure—execute a foraging trajectory and wait for reward feedback and update parameters of future trajectories—but differ in their spatial/behavioral context. Although dCA1 exhibited qualitatively similar representations across task contexts consistent with prior observations^9^, direct quantitative comparison of the encoding of spatial information, timing of SPEs and effects of optogenetic inactivation point to dissociable functional roles for dCA1 in navigational and non-navigational contexts.

The hippocampal circuit sequentially activates a sparse ensemble pattern during active behavior and ‘reactivates’ these and other potential ensemble patterns as a synchronous burst of activity (SPE)^17,52^. These SPE events are thought to be a critical window into the underlying cognitive map in hippocampus^17,19,25,26^, and it is known that SPEs can play a critical role in learning navigational trajectories via reinforcement^20–23^. However, it was not known whether SPEs play an analogous role in a non-navigational RL context in rodents, despite evidence in human subjects^19,24^. We found that SPEs were time locked to trial initiation in the non-navigational NTF task and time locked to successful trial completion in the navigational STF task, even in the same mice trained on each task. This difference in the timing of SPEs was consistent with differential effects of dCA1 inactivation at the time of SPEs in the NTF and STF tasks. In the NTF task, inactivation attenuated the initiation of target-directed forelimb movements. In the STF task, inactivation impaired updating of navigational trajectories on subsequent trials. These data provide key causal evidence for the proposed role of SWRs (here indexed by SPEs) in immediate (this trial or next) use for updating behavior^19,24,25^. This complements the now well-established, necessary role of SWRs in learning and consolidation over minutes to hours in navigational tasks^20–22^.

What might explain the difference in SPE timing and effect of dCA1 inactivation across task contexts? The initiation of a navigational trajectory involves an orienting response and locomotion initiated in a circuit thought to include (at least) superior colliculus and mesencephalic locomotor areas^63^. As might be expected from mutually exclusive actions, skilled forelimb movements are thought to be initiated via distinct cortical and subcortical areas, including the premotor and primary motor cortical regions, motor thalamus (Extended Data Fig. 9c,d) and reticular nuclei^48,61^. dCA1 projects directly to frontal (premotor) cortical areas in rodents^64^ and may, thus, be a critical node in the thalamocortical circuit dynamics^61^ underlying the initiation of goal-directed forelimb movements but not a critical node in the midbrain structures critical for locomotion initiation. Along these lines, our optogenetic perturbations replicate prior work in which disruption of dCA1 activity does not grossly disrupt performance of navigational trajectories^20,21^.

The tasks used here require a change in trajectory after a target shift that, in this case, is primarily an adjustment in trajectory amplitude. This implies a challenging problem for a computational solution in which key components of reward seeking are associated with a position in space because the sequence of place cell activity is highly overlapping across target locations. Hippocampal representations, specifically in CA1, are thought to allow for conjunctive coding of a reward location (that is, spatial position ⋂ reward^27–29^). The reward is in a constant (distal) location in our tasks, and, thus, it would seem that additional features are required in addition to a reward representation per se^28^ to provide a sufficient representation for learning in these foraging tasks. Unlike tasks that deliver reward at fixed target location(s)^27–29^ and consistent with prior work with multiple goal locations^52,65^, we did not find clear evidence for an enrichment of place fields nor enhanced decoding resolution^27^ in the vicinity of the target and little capacity to decode position during non-navigational limb movements. Moreover, inactivation of dCA1 activity around the time of target interception had no clear effect on performance in either task context, nor did it alter updating of subsequent foraging trajectories in the STF task. Taken together, our data are difficult to reconcile with a model in which a putative target or reward location code^30^ in dCA1 place cell activity is sufficient to guide navigational trajectories in our tasks and suggest that alternative, complementary representations^13^ and/or navigational strategies may be at play^66^.

Our data are, however, well-explained by a model, tML, based upon a prior model developed to explain learned changes in forelimb trajectory amplitude^35^. The tML model postulates that the parameters (heading and speed) of future navigational trajectories are updated after completion of a successful foraging trajectory. This may be consistent with prior work arguing for independent direction and amplitude encoding for forelimb movements^48,67^ and vector representations in spatial navigation problems in bats^68^ and bees^69^. Our model exploits a representation of parameters governing the generation of trajectories rather than (only) a representation of the spatial location of targets per se. We observed ‘evaluative’ SPEs that carried information about the completed trajectory and, thus, could be relevant for such a learning update in the STF task^15^. Inactivation of dCA1 at the time of trajectory completion indeed impaired the normal reinforcement-dependent updating of future trajectories in a manner well accounted for by the tML model. If this model articulated here is correct, it would suggest that SPEs participate in updating the parameters of a generative model for trajectories that may complement or further clarify previously described roles of SPEs in generalization or planning in a spatial cognitive map^17,25^.

Our study provides a computational perspective on rapid learning of trajectories that may complement spatial map-based learning models^30,66^ and could be an important component to further close the gap between existing RL model predictions and observed navigational trajectories in multiple mammalian species^39^. In a richer environment or in a distinct context, animals will also presumably use more explicitly spatial cognitive maps. Moreover, the change in trajectory could appear quite rapid in some sessions, indicating that future modeling work may gain further explanatory power by incorporating ‘meta-learning’ components, such as knowledge of changing targets or task structure more generally. In the future, we propose that it will be critical to integrate generative models of foraging trajectories (putatively with learning as in the tML model) with other representations known to be critical for navigation, such as visual landmarks, memory of locations and contexts and path integration.

## Methods

Male and female mice, typically aged 3–6 months at the time of surgery, were used in this study. Mice were housed in a reversed 12-hour light/dark cycle (lights on at 18:00) and tested in the dark phase. All procedures were approved by the Janelia Research Campus Institutional Animal Care and Use Committee and were consistent with the standards of the Association for Assessment and Accreditation of Laboratory Animal Care.

### Guide cannula implantation

**Extended Data Fig. 10:**
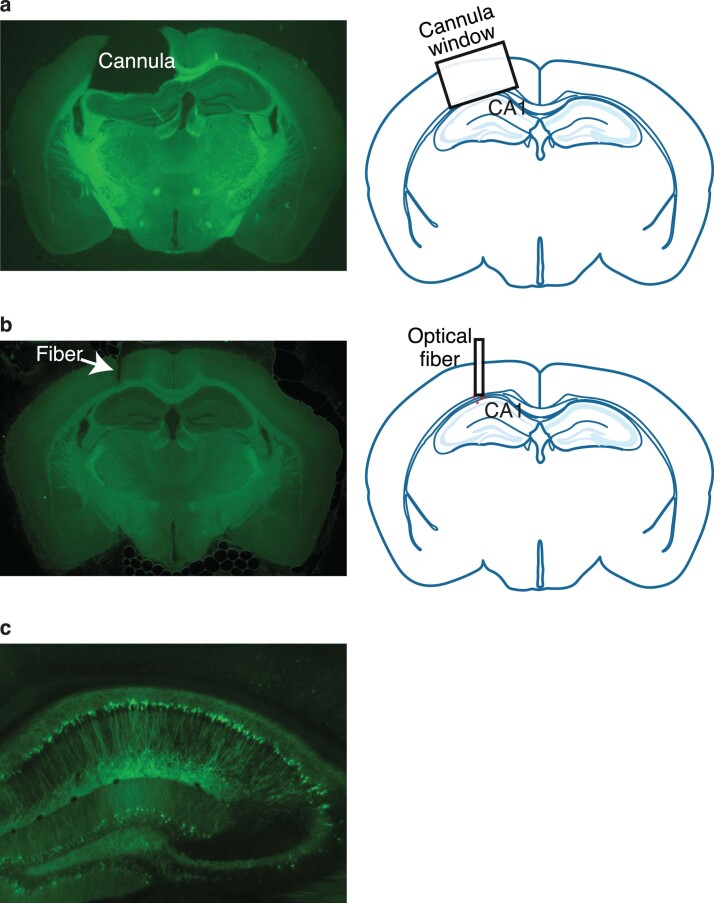
Localization of cannula, optical fibers, and GCaMP-expression. **a**, left: example histology image of cannula window for optical inhibition experiments; right: schematic of cannula positions from all experiments, which almost targeted the same position. **b**, left: example histological image of optical fiber placement; right: schematic of 4 fiber locations, whose centers are indicated by the red dots. Anatomical schematic in **a** and **b** corresponds to standard coronal atlas for bregma -1.9. **c**, Example image of GCaMP6 expression in dorsal CA1 neurons of Ai93(TITL-GCaMP6f)-D;ROSA26-ZtTA×Kcnd2-IRES-Cre 3G5 mouse.

Five male mice (three GP4.3 mice and two Ai93(TITL-GCaMP6f)-D;ROSA26-ZtTA×Kcnd2-IRES-Cre 3G5 mice) aged 3–6 months at the start were used in this study. The Kcnd2-IRES-Cre 3G5 mice were generated in house in the Janelia Transgenic Core (https://www.janelia.org/support-team/gene-targeting-and-transgenics) based upon evidence for Kcnd2 expression in principal neurons of dorsal CA1 (ref. ^71^) and are available upon reasonable request (Extended Data Fig. 10c). Mice were anesthetized under isoflurane (1.5–2%) anesthesia. A 1.8-mm-diameter circular craniotomy centered on AP −1.9 mm and ML +1.5 mm was opened with a trephine drill (1.8-mm diameter). Dura was removed, and the cortex above CA1 was aspirated with a 27-gauge blunt needle followed by a 30-gauge needle as the hippocampus was approached until vertical white fiber tracts were visible (Extended Data Fig. 10a). During this procedure, bleeding was controlled by constantly irrigating the exposed tissue with sterile 0.9% saline. Then, a guide cannula with a bottom glass window (diameter (outer): 1.8 mm and length: ~3.6 mm; part ID: 1050-002191, Inscopix) was placed above dorsal CA1. The guide cannula was affixed to the skull with dental cement (Calibra Universal Cement), and then a head bar^72^ (details can be obtained from http://dudmanlab.org/html/rivets.html) was affixed to the skull with dental cement. At the end of the surgery, the top of the guide cannula was covered by pamafilm. A silicone adhesive (Kwik-Sil, World Precision Instruments) was then applied above the parafilm.

Three to four weeks after the guide cannula implantation, awake mice were head-fixed by a head bar holder. A inner cannula lens sleeve (comes with the guide cannula; inner diameter: ~1.0 mm and length: ~4 mm) was inserted into the guide cannula first, and then a GRIN lens (1-mm diameter and ~4-mm length; part ID: 1050-002176, Inscopix) was placed into the inner cannula. A baseplate (part ID: 1050-004201, Inscopix) attached to the miniature microscope was positioned above the GRIN lens. The focal plane was adjusted until GCaMP6 fluorescence responses were clearly observed. Then, the mice were anesthetized by isoflurane, and the baseplate was affixed to the skull with dental cement.

### Optical fiber implantation and optical stimulation

VGAT-ChR2-EYFP (Jackson Laboratory, 014548, VGAT-ChR2-EYFP line 8) mice were used for optical stimulation. A guide cannula was implanted above dorsal CA1 first (same procedure as above imaging window). In the NTF task (*N* = 3 mice), at the start of each session, an optical fiber (200-mm core, 0.53 NA, doric) coupled with a 473-nm laser source (Fiberoptics) was placed into the center the guide cannula (~3-mm depth from the top of the cannula) and held by a stereotaxic micromanipulator. After each session, the optical fiber was taken out of the cannula, and the top of the guide cannula was covered by pamafilm. A silicone adhesive (Kwik-Sil) was then applied above the parafilm. In the STF task (*N* = 4 mice), an inner sleeve (~1.0-mm inner diameter, 4.0 mm long) was inserted into the guide cannula first, and then an optical fiber (200-mm core, 0.53 NA, 3 mm long, doric) was lowered down by a stereotaxic micromanipulator into the inner sleeve, until the ferrule of the optical fiber just touched the inner sleeve. The optical fiber was placed in the center of the inner sleeve. Then, dental cement was used to fix the guide tube, the inner sleeve and the ferrule of optical fiber together (Extended Data Fig. 10a).

In Extended Data Fig. 9c, a new batch of mice (*N* = 3) were trained to perform NTF tasks. Optical fibers were implanted above the hippocampus (depth: ~1 mm) in the left hemisphere and in the thalamus (depth: ~2.5 mm) in the right hemisphere. In Extended Data Fig. 8c, a new batch of mice (*N* = 4) were trained to perform STF tasks. Optical fibers were implanted above the hippocampus (depth: ~1 mm) in the left hemisphere (Extended Data Fig. 10b). During the session, optical fiber was coupled to a 473-nm laser source (Fiberoptics) to deliver light onto the dorsal hippocampus through the guide cannula window or through optical fibers directly. Then, 10-ms pulses, 25-Hz laser with power measured at the tip of the fiber of 2–3 mW, were delivered at different behavior phases with variable time length in 30% of the behavior trials. We chose this intensity to ensure complete suppression of illuminated regions of the hippocampus while minimizing effects on underlying thalamic nuclei^59^.

### Behavior: NTF task

Behavioral code was implemented as described previously and run from a microcontroller-based system (details can be obtained from http://dudmanlab.org/html/resources.html). After surgery, mice were given 5 days of recovery before beginning water restriction (1 ml of water per day). After 7 days of initial water restriction, they underwent 4–8 weeks of training. Mice were head-fixed in a custom-made head restraint box using the RIVETS head fixation apparatus^72^. The mouse’s front paws rested on a metal bar attached to a spring-loaded joystick, which had unconstrained two-dimensional maneuverability in the horizontal plane, as described previously^33,35^. Mice were trained to displace the joystick to target position ranges (as represented in figures) varying across two blocks of trials (40 trials per block) to obtain a sweetened water reward delivered 1 second after each threshold crossing. An additional lower-magnitude block was included at the end to ensure that mice were not just drifting toward larger movements over time, but these data are not shown in figures. The reward delivery was controlled by a solenoid valve outside the enclosure to minimize the audible sound of reward delivery. Rewards were followed by a 3.3-second ITI in which no movements would be rewarded. There were up to 160 trials per imaging session, with one water reward being available per trial. Forelimb movements were assessed offline to detect individual reaches based on the velocity of joystick movement. Note: NTF video data were not recorded in this dataset, but analogous performance data can be found online with a previous publication^35^.

### Behavior: STF task

After surgery, mice were given 5 days of recovery before beginning water restriction (1.2 ml of water per day). After 7 days of initial water restriction, they underwent 4–8 weeks of training in the dark phase under incandescent red light. In this self-paced free-foraging task, mice were placed in a 75 cm × 75 cm box. There was a water spout on one wall of the box (we defined an area 20 cm × 14 cm around the water spout as the collection area). Mice were required to run into an unmarked target area (~18 cm × 14 cm) triggering the reward delivery and then came back to the collection area to consume the water. The next trial starts 2 seconds after the mice enter into the collection area. See Supplementary Video 1 for an example set of trials. There were two different blocks with two different unmarked target areas: target area1 (the center is ~34 cm away from the reward area) and target area2 (the center is ~52 cm away from the reward area). The configuration of these two target areas is shown in Extended Data Figs. 1d and 3b. As the target area shifted, mice were able to reliably adjust their movements to collect rewards in both tasks. There were up to 160 trials (80 trials per block) per imaging session, with one water reward being available per trial. The reward delivery was controlled by a solenoid valve outside the enclosure to minimize the audible sound of reward delivery.

In the STF task, the mouse’s position was recorded via a USB camera mounted below the clear platform of the enclosure. In brief, a real-time tracking algorithm was developed in which the video frame was converted to black and white, subtracting a blank background without a mouse, blurred, and then a standard OpenCV blob detection algorithm was applied with user-customizable threshold settings. The center of the mouse body was calculated at every frame from the center of the detected blob, and a running buffer of positions was tracked by custom software written in Processing (www.processing.org) and written to a file. The tracking video was synchronized to the imaging using a TTL signal from the tracking program to trigger data acquisition on the Inscopix miniature microscope (www.inscopix.com). Behavior videos were linearly interpolated to match the sampling rate of the microscope. All analysis of foraging trajectories was performed offline using stored position data in MATLAB 2018 (www.mathworks.com).

### Data analysis: behavior

Real-time position data from either the joystick (NTF) or the tracked body centroid (STF) was recorded and synchronized with individual imaging frames (10 Hz or 17 Hz). The key hardware and custom software are described at http://dudmanlab.org/html/resources.html. Hardware was controlled with custom scripts written in the free software Processing and Arduino IDE (www.arduino.cc); data were analyzed with MATLAB 2020b. Occasional tracking errors were removed and interpolated over, and then *x*,*y* position data were smoothed with a Savitzky–Golay filter (3rd order, 11 pts). In the case of the STF task *x*,y position is reported relative to a reward located at *x* = 0, *y* = 0 and an arena that spanned *y* = {0,600}, *x* = {−300,300} pixel values. Video resolution was ~25 pixels for 3 cm. In the case of the NTF task, joystick position was recorded at a resolution of ~0.1 mm.

To extract trajectories, we used a custom algorithm that used a threshold amplitude (10 cm) and minimum duration (1 second) to extract trajectories and find approximate start and stop frames of individual attempts. A successful attempt was defined as one in which a reward was triggered (target intercepted) between attempt start and stop. Analogous procedures were used in the NTF task; however, movement speed profiles were more reliable metrics to use in the detection of event starts and stops. Scalar statistics of each foraging attempt (trajectory) were then computed from the positions (or derived values, angle relative to reward port, velocity, distance, etc.) between event starts and stops. For maximum amplitude, the first phase of movement was used up to a time of ~500 ms after event start to eliminate miscalculation on occasional complex trajectories that lasted for several seconds and often covered much of the environment perimeter.

### Data analysis: calcium imaging

In the NTF task, mice (*N* = 5) were head-fixed in a custom-made head restraint box using the RIVETS head fixation apparatus, and the microscope was connected to the baseplate when the animal was head-fixed; after adjusting to the best imaging focal plane, the imaging session started. In the STF task (*N* = 3 mice), first, the microscope was connected to the baseplate when the animal was head-fixed; after adjusting to the best focal plane, mice were removed from head fixation and put into the free-moving behavior box. Two mice underwent imaging in both NTF and STF tasks.

Fluorescence images were acquired at 10 Hz (GP4.3 mice) or 17 Hz (Ai93(TITL-GCaMP6f)-D;ROSA26-ZtTA×Kcnd IRESCre 3G5 mice; www.jax.org), and the LED power was set at 10–35% (0.1–0.35 mW) with analog gain of 3–3.5. To decrease the photo bleaching effect, in each session imaging trial segments were interleaved with non-imaging segments. During the imaging trial segment, the LED in the microscope was on, and GCaMP signals were acquired; whereas, during the non-imaging segment, the LED and imaging acquisition were off, but the behavior task still kept going. In the NTF task, 20 trials were recorded in each block. In the STF task, 40 trials were recorded in each block. All recorded calcium videos from one animal in 1 day were concatenated in Fiji. The concatenated video was spatially downsampled 2× and movement-corrected using Mosaic (www.inscopix.com). Then, the corrected video was cropped to remove correction artifacts and exclude areas with no GCaMP6f^42^ activity. The cropped video was further spatially downsampled 2× (usually resulting in 350 × 300 pixel videos). The CNMF-E package^73,74^ was used to automatically segment neurons from the pre-processed videos. The neuron ROIs from CNMF-E were manually examined and corrected. Calcium signals within these corrected ROIs were extracted with CNMF-E. Spike trains were inferred with the deconvolution function in the CNMF-E package (constrained FOOPSI).

To align neurons from different days, MATLAB scripts based on the ANTs toolkit (http://picsl.upenn.edu/software/ants/) were used to register images from different days and generate the corresponding transformations. Using these transformations, the neuron ROIs of different days were transformed to align with a common reference ROI. Then, pairwise correlation coefficients of spatial profiles of the transformed ROIs across days were computed. If the correlation coefficient was greater than 0.8, this pair of ROIs was assigned as the same neuron on different days.

### Data analysis: place fields

To analyze place fields, we identified ‘movement periods’ when the mouse ran in open-field arenas at the speed of ≥1 cm s^−1^. These criteria rejected small movements, such as grooming, rearing or head turning. We spatially binned the open-field arenas into 4 cm × 4 cm bins. To suppress noise, we also identified ‘foraging bins’, into which bins the mouse ran ≥5 times in one session. We divided the number of calcium transients in each foraging bin by the mouse’s total occupancy time there, applied a Gaussian smoothing filter (σ = 4 cm) and normalized each place field by its maximum value.

### Data analysis: neural correlates of behavior

In brief, in both tasks, individual movements in trained mice were quite well-isolated (see extended traces in Figs. 1 and 3). In the NTF task, analysis was preceded by identifying the start and stop time of each individual movement. Movements were required to be at least 1 second in duration with at least 1 second between well-separated movements. Raw position data were centered around either the reward collection port (STF task) or the true 0 position of the joystick (NTF task). Speed was computed by taking a simple pointwise difference and smoothing with a Savitzky–Golay filter. In the NTF task, a threshold was used to estimate the onset and offset of movement events. A number of statistics of movement were then computed from these events. Whether a movement event was rewarded or not was determined by looking for reward triggers occurring during an event. More than 95% of rewards could be attributed to a single well-isolated movement event in all sessions used for analysis.

Cross-validated PMTH alignment in Fig. 3 was determined by taking a random half of trials, sorting by time of peak response magnitude and then using that ROI index array to sort the held-out half of trials. The results were plotted in Fig. 3 for both tasks. For continuous plots of data shown in Fig. 3, we accomplished a hierarchical sorting of activity by first dividing ROIs around the median of average activity over the session and then, within each group, re-sorting by latency to peak response from movement onset. This array of ROI indices was used in all subsequent plotting.

In Fig. 5 and Extended Data Fig. 7, we calculated the principal components of population activity or specifically around the period of time during which SPEs occurred to bias toward variance in ensemble patterns during SPEs. In the latter case, we detected SPEs as described in Extended Data Fig. 5, took brief windows (±250 ms; similar results with ±125 ms, not shown) of activity (Δ*F*/*F*) and concatenated all SPE-triggered windows into a single matrix. We then computed the principal components of this matrix where the leading component is denoted as ‘PC^SPE^’.

### Data analysis: decoder and classifier construction

To decode the continuous behavior from inferred spikes in the imaging data, we took an approach we recently described^48^ that is inspired by the use of committee machines in machine learning. In brief, we sought to identify a linear decoder to estimate the joystick movement or body position. The decoder defines linear mapping (*W*_*decode*_) between the neural population activity and the two-dimensional position:

$$K = F^T \cdot W_{decode}$$

where *F* is the data matrix comprising the population vector of spike counts with the dimension of the number of units concatenated across all time bins and trials in the training dataset. The matrix *K* comprises two vectors each corresponding to the decoded position ({*x*,*y*} or {angle,radius}). We solve for *W*_*decode*_ as *W*_*decode*_ = (*F*^*T*^*F*)^*^F^*T*^*K* using the Moore–Penrose inverse on a subset of randomly permuted and concatenated trials. This approach yielded noisy decoder performance on cross-validation. To reduce noise and provide better generalization, we computed a family of linear decoders from N folds of P permuted trials. Typical values were N = 50 and P = 75. We then took the mean of the family of decoders (*N* × number of units) to yield a ‘consensus’ decoder. Decoding performance is illustrated with this consensus decoder applied to a unique permuted sequence of trials; see also ref. ^48^.

### Data analysis: perturbation effects on STF task

To assess the effects of optogenetic inactivation of hippocampal neuronal ensemble on STF task, we extracted each individual foraging trajectory run as described above. We then identified runs that contained a laser perturbation either in the collection area immediately after run termination or during interception of the target area. For each run trajectory, we then computed its maximal amplitude and initial angle and calculated the change (delta) in amplitude and heading angle relative to the prior trial. The analyses conditioned each delta for trial *i* on whether trial *i*−1 was unrewarded, rewarded or reward+laser inactivation and took the session-wise mean for each of the three conditionals. Box plots shown in the figures represent the distribution of session-wise mean effects across animals and sessions. Significance testing was performed by calculating the Kruskal–Wallis test and reporting *P* values. Multiple comparison corrections for repeated measures from individual mice were performed using the kruskalwallis function in MATLAB and the returned ANOVA table. The same analysis approach was used for all perturbation types and for the analysis of tML model trajectories (see below). Reported *P* values are for the main effect of prior trial type (unrewarded, rewarded and perturbed) with correction for multiple comparisons.

### Data analysis: perturbation effects in NTF task

To assess the effects of optogenetic inactivation of hippocampal neuronal ensemble on NTF task, forelimb movements were aligned with the reward event (Fig. 7b) within 12 seconds after that event. Because only ~30% of trials were inactivation catch trials, we randomly resampled (with replacement) *k* trials of the aligned movement from the catch and control trials, respectively, where *k* is the number of catch trials. Then, we used the aligned movement in the resampled catch and control trials to compute their post-event time histograms (PETHs) of movement. To statistically evaluate the difference in PETHs between catch and control trials, we repeated the resampling and PETH calculation procedure 1,000 times. Mean PETHs and 95% (2.5–97.5%) confidence intervals (CIs) of PETHs under inactivation and control conditions were calculated with the 1,000 resampled PETHs. To remove transient noise, only the time spans greater than 200 ms and no overlapping between the 95% CIs were marked with red horizontal lines in Fig. 7b to show when inactivation significantly affected NTF behavior.

### Computational modeling: tML agent

The tML agent model is based around the idea that an agent can learn to scale the parameters of a structured representation of foraging trajectories. For a central place forager, we might demand a trajectory that forms a closed out-and-back loop that begins at a ‘home’ location, transits through an extrema and returns home. The goal of the learning agent is to update the heading and amplitude of this trajectory so that it reliably intercepts a target location according to the specific rules of the environment. For example, interception may need to occur at the trajectory extrema or perhaps anywhere along the trajectory or perhaps for some fixed duration. In the specific cases for this study, we consider interception at any point along the trajectory (STF task) and for a fixed, brief duration (NTF task) that correspond to the practical requirements of our real-time behavior analysis used in the experimental task designs. We note that similar results to those reported have been obtained with a range of different simulated environments.

Returning to the notion of a structured, closed-loop trajectory, we consider the problem as a control signal that determines behavior at each time step. First, considering a locomoting animal, at each time step we assume that it is governed by a heading angle and an instantaneous speed. Under such a model, a closed-loop trajectory will be produced by a smooth rotation in heading angle (a linear function from −pi to pi). For a fixed speed, this would produce a rotation about a circle. However, to produce the observed, roughly elliptical paths, speed is inhomogeneous and reaches maxima along specific heading angles—outward runs (pi/2) and return runs (−pi/2). Given the expected bell-shaped distribution of speeds that minimize jerk along a trajectory, this can be modeled as a sequence of Gaussian speed profiles. These dynamics for heading and speed can be generated by an artificial neural network, but, for simplicity, we have used simple generative functions perturbed by noise. A schematic of the model architecture can be found in Figs. 2, 6 and 7.

1 $${\Theta}(t) = L( - \pi ,\pi ) + \omega [i] + \varepsilon$$

2 $$S(t) = G(tau,\sigma ) \times a[i] + \varepsilon$$

where ***L*** is a linear mapping across the range {−*π*, *π*} spanning time *t*, and *ω*[*i*] is the heading offset sampled from a distribution of mean Ω[*i*] and constant variance for each trial *i*. *G* is either a single (NTF) or double (STF) peaked Gaussian function with offsets $tau = \{ tau1\} \,or\,\{ tau1,tau2\}$ and width *σ*, scaled by a gain *a*[*i*] sampled from a distribution with a mean *A*[*i*] and constant variance for each trial *i*. *ε* is a normally distributed and smoothed noise term matched to observed variability in observed behavioral trajectories.

Given a model of structured trajectories defined by continuous speed (*S*(*t*)) and heading (*θ*(*t*)), the learning problem for an agent is to learn to scale the amplitude (*A*(*i*)) and orientation (Ω(*i*)) offsets of trajectories trial by trial to reliably intercept target locations. Behavioral data indicated bidirectional and rapid learning for changes in the scaling of movement trajectories; thus, we used a modified version of a learning rule (MeSH) previously described to account for rapid, bidirectional movement scaling^35,40^.

3 $$\left. {A[i + 1] = A[i] + \alpha (a[i] - A[i])\upsilon [i] - \beta (a[i] - A[0])} \right)$$

4 $$\left. {{\Omega}[i + 1] = {\Omega}[i] + \alpha \left( {\omega [i] - {\Omega}[i]} \right)\upsilon [i] - \beta \left( {\omega [i] - {\Omega}[0]} \right)} \right)$$

where *i* is the index of the *i*th trial; $\upsilon [i]$ is a smoothed estimate of the local reward rate; and *a*[*i*] is the magnitude of the speed on the current trial as sampled from a normal distribution centered on *A*[*i*] with rate parameters *α* and *β*. Learning rate parameters and the standard deviation of the distribution ${{{\mathcal{N}}}}(A,\sigma )$ were explored using grid search optimization. The equivalent learning rule is also expressed for Ω(*i*) in Eq. 4.

To account for effects of inactivation, we considered two implementation modifications to the tML model corresponding to the distinct behavioral context of the STF and NTF tasks (Discussion). Simulation data and schematics depicting these model formulations are shown in Table 1 and Figs. 6 and 7.

First, we consider the STF task in which CA1 SPEs were observed immediately upon completion of a foraging trajectory and return to the reward location. The critical computation for learning in the tML model is the MeSH update (Eq. 3) that depends upon the signed difference between the current trial speed, *a*[*i*], (or heading) and the current policy speed, *A*[*i*], (or heading). A precise circuit mechanism for this computation is unclear and beyond the scope of the current study, but one possibility consistent with our experimental data is that CA1 SPEs encode information about the current trajectory. In such a formulation, we consider a model in which the SPE is necessary to update the policy, and, in the absence of an SPE, the policy reverts to its default *A*[0].

Second, we consider the NTF task in which CA1 SPEs were observed just before initiation of a joystick movement. Again, we postulate that the occurrence of an SPE is critical for a learning update; however, we note some key differences in the control of skilled forelimb movements as contrasted with navigational trajectories (Discussion). Previous modeling work in the context of tasks like the NTF task have been consistent with the possibility that the putative MeSH update is produced in the form of an eligibility trace at the time of movement initiation^35,40^. Here, we consider the additional possibility that movement initiation is facilitated by the occurrence of a CA1 SPE. We note that this would be a particularly useful formulation to ensure that a viable eligibility trace is present when movements are initiated given the width of the distribution of movement initiation times and relatively low frequency of SPEs of about 1 Hz. To model such an initiation process, we generated a hazard function that matched the observed latency distribution and determined the probability of initiating a trial. The hazard function is given by:

$$H = g(\mu ,\sigma )/1 - G$$

where *g* is a Gaussian function with mean of 3 seconds and standard deviation of 0.48 seconds. *G* is the cumulant density of *g*. Individual trial latencies were determined by sampling a uniform random variable for the timepoint at which it exceeded probability *H*, if an SPE had occurred. We used the observed empirical distribution of intervals between SPEs for all datasets to draw event times for an SPE. In the case of optogenetic inactivation, we assume that the probability of an SPE was reduced by ~75% but also resulted in an SPE with high probability at offset of inactivation due to rebound excitation^58^.

For simulations of a standard Q agent (Extended Data Fig. 1e,f) to examine exploration around a target switch, we assumed a converged, optimal value estimate and simulated trajectories using a standard ε-greedy simulated agent^36^. For shown simulations, ε = 0.1, 0.4 or 1 (that is, random walk agent). Notes: (1) Qualitatively similar results are obtained by training models to criterion; however, it requires large numbers of trials that exceed total experience of mice; (2) The simulations are not the full task and do not have a mechanism for returning to reward collection location. This has not, to our knowledge, previously been modeled, and it is neither clear how it should be implemented nor whether it continues to exhibit the optimal convergence properties that make Q learning attractive in the first place, because some switching dynamics of the action–value function are implied; and (3) A deterministic Q agent with an optimal value function never obtains reward after a target switch and, thus, is not shown.

### Reporting summary

Further information on research design is available in the Nature Portfolio Reporting Summary linked to this article.

## Online content

Any methods, additional references, Nature portfolio reporting summaries, source data, extended data, supplementary information, acknowledgements, peer review information; details of author contributions and competing interests; and statements of data and code availability are available at 10.1038/s41593-022-01201-7.

### Supplementary information

Reporting Summary

Supplementary Video 1 Example video of the STF task control and experimenter view

## Data Availability

The imaging data used in this manuscript will be made available at https://janelia.figshare.com/; 10.25378/janelia.21539676. A compiled set of links to data, supporting files, hardware information, and code can be found at https://tinyurl.com/Wanchen2022.

## Code availability

Code for running simulations in this study is available at https://github.com/dudmanj/tML and/or http://dudmanlab.org upon publication.
