# Supplementary material for: Hippocampal representations of foraging trajectories depend upon spatial context
Source: Nat Neurosci. 2022 Nov 29;25(12):1693–705. doi: 10.1038/s41593-022-01201-7 (PMC9708565; doi:10.1038/s41593-022-01201-7)
Supplement: Supplementary file 1 — Reporting Summary [file 41593_2022_1201_MOESM1_ESM.pdf]

nature portfolio

Corresponding author(s): Dudman, Jiang

Last updated by author(s): Oct 11, 2022

Reporting Summary

Nature Portfolio wishes to improve the reproducibility of the work that we publish. This form provides structure for consistency and transparency in reporting. For further information on Nature Portfolio policies, see our Editorial Policies and the Editorial Policy Checklist.

Statistics

For all statistical analyses, confirm that the following items are present in the figure legend, table legend, main text, or Methods section.

n/a	Confirmed	
☐	☒	The exact sample size (*n*) for each experimental group/condition, given as a discrete number and unit of measurement
☐	☒	A statement on whether measurements were taken from distinct samples or whether the same sample was measured repeatedly
☐	☒	The statistical test(s) used AND whether they are one- or two-sided<br>*Only common tests should be described solely by name; describe more complex techniques in the Methods section.*
☒	☐	A description of all covariates tested
☐	☒	A description of any assumptions or corrections, such as tests of normality and adjustment for multiple comparisons
☐	☒	A full description of the statistical parameters including central tendency (e.g. means) or other basic estimates (e.g. regression coefficient) AND variation (e.g. standard deviation) or associated estimates of uncertainty (e.g. confidence intervals)
☐	☒	For null hypothesis testing, the test statistic (e.g. *F*, *t*, *r*) with confidence intervals, effect sizes, degrees of freedom and *P* value noted<br>*Give P values as exact values whenever suitable.*
☒	☐	For Bayesian analysis, information on the choice of priors and Markov chain Monte Carlo settings
☐	☒	For hierarchical and complex designs, identification of the appropriate level for tests and full reporting of outcomes
☐	☒	Estimates of effect sizes (e.g. Cohen's *d*, Pearson's *r*), indicating how they were calculated

Our web collection on statistics for biologists contains articles on many of the points above.

Software and code

Policy information about availability of computer code

Data collection	Data was collected using behavioral paradigms and hardware described on our lab website dudmanlab.org and miniscopes and acquisition and analysis software from Inscopix.
Data analysis	All data was analyzed using Matlab (checked with versions 2019a-2021b). Code is available at the corresponding GitHub repository for this manuscript https://github.com/dudmanj/tML

For manuscripts utilizing custom algorithms or software that are central to the research but not yet described in published literature, software must be made available to editors and reviewers. We strongly encourage code deposition in a community repository (e.g. GitHub). See the Nature Portfolio guidelines for submitting code & software for further information.

Data

Policy information about availability of data

All manuscripts must include a data availability statement. This statement should provide the following information, where applicable:

- Accession codes, unique identifiers, or web links for publicly available datasets
- A description of any restrictions on data availability
- For clinical datasets or third party data, please ensure that the statement adheres to our policy

Data created for this manuscript will be publicly available at janelia.figshare.com - https://doi.org/10.25378/janelia.18314717

Human research participants

Policy information about studies involving human research participants and Sex and Gender in Research.

Reporting on sex and gender	N/A
Population characteristics	N/A
Recruitment	N/A
Ethics oversight	N/A

Note that full information on the approval of the study protocol must also be provided in the manuscript.

Field-specific reporting

Please select the one below that is the best fit for your research. If you are not sure, read the appropriate sections before making your selection.

☒ Life sciences ☐ Behavioural & social sciences ☐ Ecological, evolutionary & environmental sciences

For a reference copy of the document with all sections, see nature.com/documents/nr-reporting-summary-flat.pdf

Life sciences study design

All studies must disclose on these points even when the disclosure is negative.

Sample size	Sample sizes were chosen based upon previous work using similar measurements and experimental paradigms - e.g. https://www.nature.com/articles/nn.3329 and generally in the field of hippocampus/spatial foraging reviewd in https://www.nature.com/articles/s41583-021-00479-z
Data exclusions	Data were not excluded, but some sessions were stopped early or not recorded if the animal exhibited limited behavior or imaging was deemed to be a low quality or any potential health indications as assessed by vivarium staff and according to our IACUC protocol.
Replication	Experiments were performed without specific blinding to condition; however, in optogenetic perturbation experiments interleaved trials were selected for perturbation automatically in a pseudorandom manner by the control software. All behavior was automated and the experimenter was not directly engaged in running the tasks directly. As described in the text multiple sessions (>5 replicates) we used for individual animals. We show results from inidividual sessions throughout.
Randomization	Randomization of trials for perturbation was programmed pseudorandomly (uniform random probability with bounds preventing long runs of perturbation trials). Otherwise only a single condition was examined and thus randomization was not required.
Blinding	Experiments were performed without explicitly blinding to condition. In practice measures were takent o prevent accidential knowledge or bias by automating the entire experimental procedure. Selection of sessions with good imaging data quality were chosen without regard to or knowledge of neural correlates to behavior or other potentially biasing information.

Reporting for specific materials, systems and methods

We require information from authors about some types of materials, experimental systems and methods used in many studies. Here, indicate whether each material, system or method listed is relevant to your study. If you are not sure if a list item applies to your research, read the appropriate section before selecting a response.

Materials & experimental systems

n/a	Involved in the study
☒	☐ Antibodies
☒	☐ Eukaryotic cell lines
☒	☐ Palaeontology and archaeology
☒	☐ Animals and other organisms
☒	☐ Clinical data
☒	☐ Dual use research of concern

Methods

n/a	Involved in the study
☒	☐ ChIP-seq
☒	☐ Flow cytometry
☒	☐ MRI-based neuroimaging
